# Inhibitors of PD-1 in Non-Small Cell Lung Cancer: A Meta-Analysis of Clinical and Molecular Features

**DOI:** 10.3389/fimmu.2022.875093

**Published:** 2022-04-05

**Authors:** Gengwei Huo, Wenjie Liu, Peng Chen

**Affiliations:** ^1^Department of Thoracic Oncology, Tianjin Medical University Cancer Institute and Hospital, National Clinical Research Center for Cancer, Key Laboratory of Cancer Prevention and Therapy of Tianjin, Tianjin’s Clinical Research Center for Cancer, Tianjin, China; ^2^Department of Oncology, Jining No. 1 People’s Hospital, Jining, China

**Keywords:** PD-1 inhibitors, potency, predictor, non-small cell lung cancer, meta-analysis

## Abstract

**Objective:**

PD-1 inhibitors have become an indispensable treatment in Non-Small Cell Lung Cancer (NSCLC), but the potential predictive value of clinical and molecular features need to be clarified. The objective of the study was to study the potency of PD-1 inhibitors in patients with NSCLC in contexts of both clinical and molecular features, and to aid identification of patients for choice of type of PD-1 inhibitor therapy in order to acquire more accurate NSCLC treatment in immunotherapy.

**Method:**

PubMed, Google Scholar, Embase Science Direct, the Cochrane library, and major oncology conferences were searched for randomized clinical trials (RCTs) that were published prior to December 2021. RCTs that had PD-1 inhibitor alone or in combination with chemotherapy with non-PD-1 inhibitor for the treatment of NSCLC patients were selected. Two authors independently selected studies, data extraction and bias risk assessment. Basic characteristics of included studies, and also the 95% confidence interval and hazard ratios of the overall patients and subgroups were recorded. The inverse variance weighted method was used to estimate pooled treatment data.

**Result:**

A total of eleven RCTs including 5,887 patients were involved. PD-1 inhibitors-based therapy substantially enhanced OS compared with non-PD-1 inhibitor therapy in patients with age group <65 years, 65–74 years, active or previous smokers, without brain metastases, liver metastases, EGFR wild-type patients, individuals in East Asia and U.S./Canada, but not in patients with age group ≥75 years, never smokers, brain metastases, EGFR mutant patients or individuals in Europe. OS was improved in patients with NSCLC who received PD-1 inhibitors regardless of their gender (male or female), histomorphological subtypes (squamous or non-squamous NSCLC), performance status (0 or 1), and PD-L1 tumor proportion score (TPS) (<1%, ≥1%, 1–49%, or ≥50%). An analysis of subgroups revealed that, patients with age group <65 years old, male, non squamous cell carcinoma, PS 1, TPS ≥1%, and TPS ≥50% benefited from pembrolizumab treatment not related with treatment line and treatment regimen.

**Conclusion:**

Age group, smoking history, metastasis status/site, EGFR mutation status, and region can be used to predict the potency of PD-1 inhibitors, and to be individualized to choose different types of PD-1 inhibitors, and treatment regimen for NSCLC patients.

## Introduction

Lung cancer is one of the most common lethal solid malignancies and the leading cause of death worldwide (1). Non-small cell lung cancer (NSCLC) accounts for almost 85% of all lung cancers in histology (2). During the past two decades, studies in immunobiology and the immune checkpoint-blockade therapy of cancers have stimulated further interests in immunotherapy of NSCLC (3–5). Immune checkpoint inhibitors (ICIs) have become a 1st-line treatment in a variety of malignant tumors, adding immunotherapy to the ranks of surgery, chemotherapy, radiotherapy and targeted therapy (6, 7). So far, the outcome of many large-scale randomized controlled trials (RCTs) of PD-1 inhibitors against NSCLC individuals have verified the concept of lasting anti-tumor response and improved progression free survival (PFS) and overall survival (OS) (8).

However, only a minority of individuals have benefited from PD-1 inhibitors (9), and it becomes even more urgent to investigate suitable biomarkers in order to identify individuals who are candidates for PD-1 inhibitor therapy and to achieve accurate treatment of NSCLC—both to protect individuals from ineffective treatments and to limit the number of individuals exposed to potential autoimmune side effects from drugs targeting the axis (10, 11).

To date, the best-known and most commonly used biomarker is the expression of PD-L1 in NSCLC, as detected by immunohistochemistry. PD-1 inhibitor therapy is more likely to benefit patients who have high levels of PD-L1 expression reflected in tissue samples (12, 13). Unfortunately, tissue samples are not only difficult to obtain, but are very small in size. Furthermore, the lack of unification between various anti-PD-L1 clones and immunohistochemistry platforms is also an intractable issue (11, 14, 15). Another predictive biomarker is tumor mutation burden (TMB) assessed even from cell blocks (16), but there was no consensus. The KEYNOTE-158 found better response rates of pembrolizumab in patients with high tissue TMB (17), while KEYNOTE-021 and KEYNOTE-189 did not demonstrate a strong correlation between TMB and PD-1 inhibitor potency (18, 19). In addition, microsatellite instability (MSI) and other emerging biomarkers, although promising, also have some limitations (15, 20, 21).

It is of great significance to search other economic and practical factors for predicting the potency of PD-1 inhibitors. There are differences in the role of PD-1 inhibitors among individuals with varying clinical and molecular features (22). As a result, we performed this meta-analysis to determine the predictive value of various clinical and molecular attributes for guiding the selection of individuals with NSCLC who should benefit from PD-1 inhibitors. We provide the following article based on the PRISMA reporting checklist.

## Methods

### Inclusion and Exclusion Criteria

The selection of studies that met the inclusion and exclusion criteria was based on the elements of the PICOs (participants, intervention, comparison, and outcomes), with each letter representing the components as population of patients (P), articulation or interventions (I), the comparator/reference group (C), the outcome (O), and the design of the study (S). Prior to screening studies by title and abstract, duplicate articles were removed from the gathered studies. This was done in order to identify research papers that fulfilled the following inclusion criteria: (I) PD-1 inhibitor alone or in combination with chemotherapy compared with non-PD-1 inhibitor for the treatment of NSCLC individuals, (II) reported hazard ratio (HR) and confidence interval (CI) 95% for progression free survival (PFS) and/or overall survival (OS) with predefined subgroups, such as age group, gender, histomorphological subtypes, Eastern Cooperative Oncology Group (ECOG) performance status (PS) score, smoking status, metastasis status/sites, EGFR mutation status, region, and PD-L1 tumor proportion score (TPS), (III) multiple studies confirmed the same trial, utilizing the most recent data with the largest patient population and the longest follow-up, (IV) numerous articles described distinct subgroups of the same trial; we incorporated them all.

The following exclusion criteria apply to a study that is discovered (I) without distinguishing between the effects of multiple PD-1 inhibitors, and has (II) insufficient survival data available or the control group garnered only a placebo. For the information resources, we consulted not only the full text of the article, but also the appendix and the references listed at the end of each article.

### Literature Survey and Data Collecting

Our search terms and medical subject headings were specific enough that we were able to find results in a variety of electronic databases, namely, PubMed, Google Scholar, Embase Science Direct, the Cochrane library, and also the proceedings of major oncology conferences. The major browse terms were non-small cell lung cancer, PD-1 inhibitors, predictor, potency and randomized controlled clinical trial, which were supplemented with several other terms, but may not be restricted to pembrolizumab, nivolumab, clinical and molecular characteristics. The search was restricted to research articles that were published prior to December 2021, according to the search criteria. In addition, bibliographies of significant related articles were screened for inclusion in the database.

Two authors (WL and GH) independently selected studies and extracted data from those studies. They went through all of the studies and determined whether or not they were eligible based on the previously described inclusion criteria. If there were any disagreements, the third author would be consulted (PC). Each study provided the following information: the title of the study, 1st author, and year of publication, gender distribution, the mean age group, the design and blinding of the study, study phase, line of therapy, study drug, and the survival outcome measures of predefined subgroups.

### Quality Assessment and Statistical Analyses

The validity and reliability of the study was evaluated by two researchers who worked independently (WL and GH) using the Cochrane Bias tool. We performed all the statistical analysis using the statistical software Review Manager 5.3. The primary endpoint of the study was to compare OS in the PD-1 inhibitor treatment group and the non-PD-1 inhibitor group, which was measured by HR and corresponding CI. PFS was used as a secondary endpoint in this experiment. The HR was calculated using either random-effects or fixed-effect models, depending on the heterogeneity of the studies included in the analysis. The existence of heterogeneity was tested using the Chi-square test and I^2^ statistics test. If heterogeneity was considered acceptable (I^2^ <50% and P >0.10), a fixed-effect model was utilized; otherwise, the random effect model was utilized. Due to the fact that the treatment of interest is typically evaluated in a single trial, fixed-effect models are employed. The results are presented as forest plots, along with pooled summary estimates and the 95% CI that correspond to these estimates. The logarithmic scales on forest plots were used to manually extract HRs and 95% CIs when they were not directly reported by the authors in the text. Sensitivity analysis was performed by excluding studies with a small sample size or studies for which the HR and associated 95% CI could not be obtained directly from the studies themselves. The nominal level of significance was set at P <0.05.

## Results

### Study Selection and Characteristics

**Figure 1:**
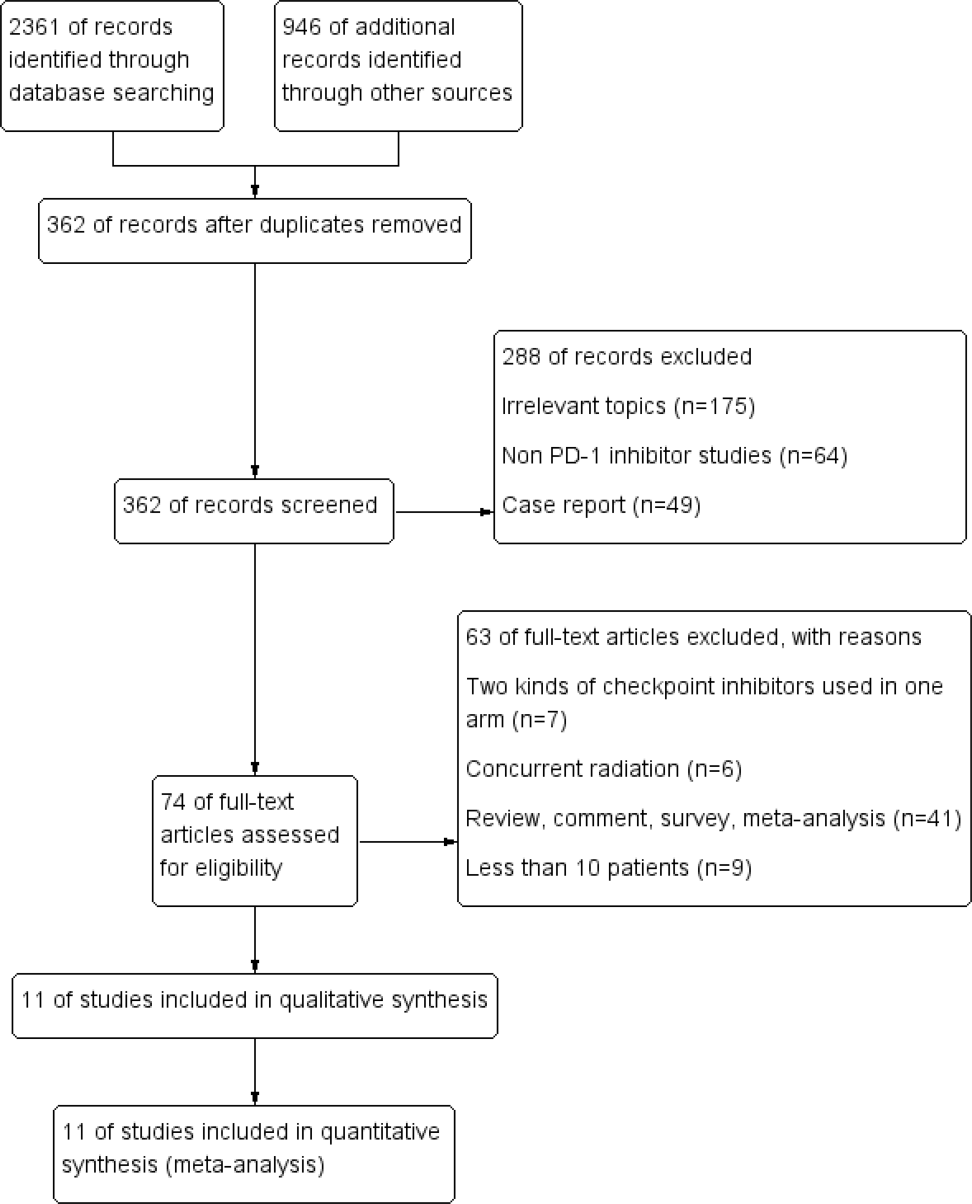
PRISMA flow diagram.

**Figure 2:**
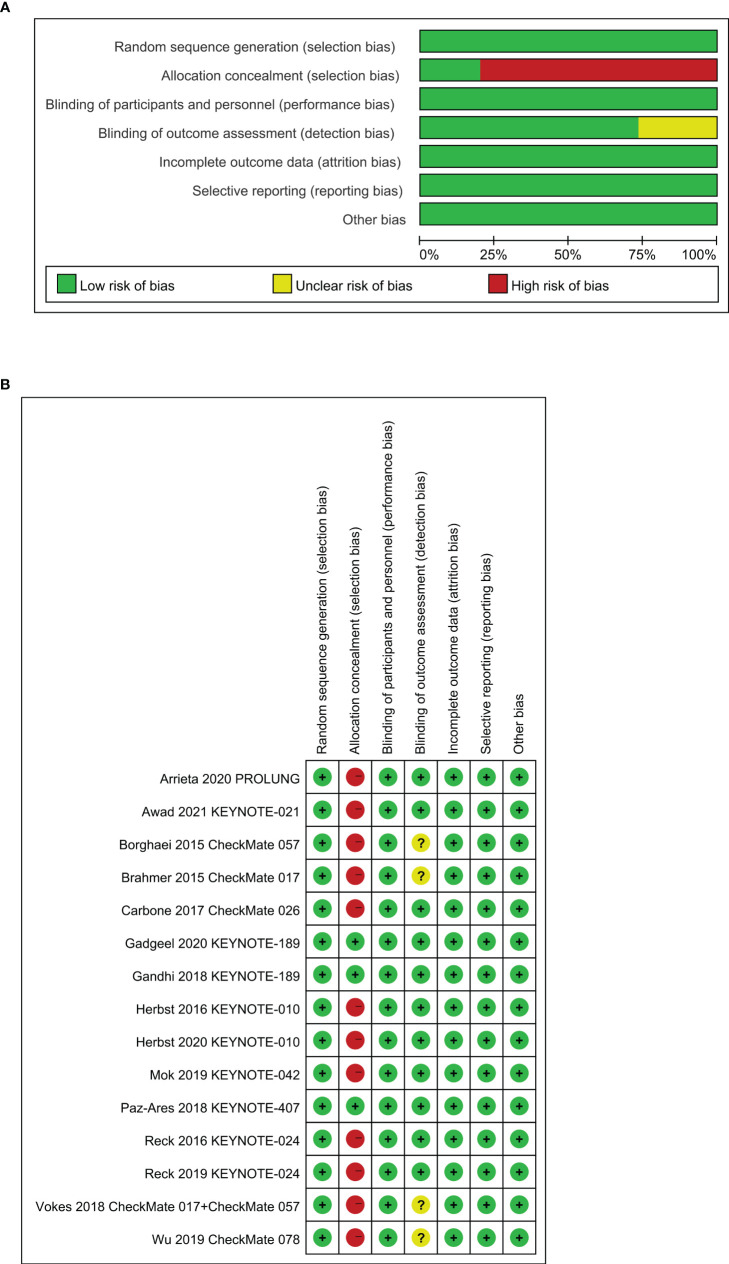
Assessment of bias risk, **(A)** risk of bias graph, **(B)** risk of bias summary.

An estimated 3,307 potentially relevant records were identified from databases and conferences as a result of the search strategy employed in the research. **Figure 1** depicts the selection process and the rationale for excluding studies that were deemed ineligible. A total of 3,296 studies were excluded after they were screened for their abstracts and full texts. Thus, 11 randomized controlled trials (RCTs) involving 5,887 patients with advanced NSCLC were considered for inclusion in the meta-analysis (**Table 1**). These clinical trials were published between 2015 and 2021 and were divided into the following categories: Two of the studies were clinical trials in the II phase (23, 24), one was phase II/III trial (31, 32), and eight were phase III trials (25–30, 33–37). Particularly notable is that, despite the fact that KEYNOTE-407 released updated potency data in 2020, there was no data on eligible subgroup analyses, and as a result, it was excluded from the meta-analysis (38). The detailed risk of bias analysis revealed that there was a low risk of bias in all RCTs (**Figure 2**).

**Table 1:** Basic characteristics of included studies.

Reference	Trial	Year	Study phase	Stage	Treatment line	ICI used (n)	Control arm (n)	Median (range) Age (years)	Males (%)	Never smokers (%)	Squamous (%)	Nsquamous (%)	Tumor PD-L1 expression			ECOG		Primary endpoint
													<1% (%)	≥1% (%)	Unknown (%)	0 (%)	1 (%)	
Arrieta et al. (23)	PROLUNG	2020	II	Advanced/metastatic	≥2L	Pembrolizumab + docetaxel (40)	Docetaxel (38)	60 (27–78)	41	43.6	0	89.7	38.5	38.5	23.0	14	86	ORR
Awad et al. (24)	KEYNOTE-021	2021	II	IIIB/IV	1L	Pembrolizumab + pemetrexed + carboplatin (60)	Pemetrexed + carboplatin (63)	64.3 (37–80)	39	20	0	100	36	64	0	43	56	ORR
Borghaei et al. (25)	CheckMate 057	2015	III	IIIB/IV/recurrence/progression	≥2L	Nivolumab (292)	Docetaxel (290)	62 (21–85)	55	20	0	100	35.9	42.3	21.8	31	69	OS
Vokes et al. (26)		2018																
Brahmer et al. (27)	CheckMate 017	2015	III	IIIB/IV/recurrence/progression	2L	Nivolumab (135)	Docetaxel (137)	63 (39–85)	76	6	100	0	39.0	43.7	17.3	24	76	OS
Vokes et al. (26)		2018																
Carbone et al. (28)	CheckMate 026	2017	III	IV/recurrence	1L	Nivolumab (271)	Platinum doublet chemotherapy (270)	64 (29–89)	61	11	24	76	0	100	0	33	66	PFS
Gandhi et al. (29)	KEYNOTE-189	2018	III	metastatic	1L	Pembrolizumab + pemetrexed + platinum (410)	Placebo + pemetrexed + platinum (206)	64.5 (34–84)	58.9	11.9	0	100	30.8	63.0	6.2	43.2	56.0	OS and PFS
Gadgeel et al. (30)		2020																
Herbst et al. (31)	KEYNOTE-010	2016	II/III	IIIB/IV	≥2L	Pembrolizumab (690)	Docetaxel (343)	62.7 (56–69)	61.4	18.4	21.5	70.3	0	100	0	33.6	65.7	OS and PFS
Herbst et al. (32)		2020																
Mok et al. (33)	KEYNOTE-042	2019	III	Advanced/metastatic	1L	Pembrolizumab (637)	Platinum-based chemotherapy (637)	63 (57–69)	70.8	22.1	38.6	61.4	0	100	0	30.6	69.4	OS
Paz-Ares et al. (34)	KEYNOTE-407	2018	III	IV	1L	Pembrolizumab + paclitaxel/nab- paclitaxel + carboplatin (278)	Placebo +paclitaxel/nab-paclitaxel + carboplatin (281)	65 (29–88)	81.4	7.3	97.7	2.3	34.7	63.2	2.1	29.2	70.8	OS and PFS
Reck et al. (35)	KEYNOTE-024	2016	III	IV	1L	Pembrolizumab (154)	Platinum-based chemotherapy (151)	65.2 (33–90)	61.3	7.9	18.4	81.6	0	100	0	35.1	64.6	PFS
Reck et al. (36)		2019																
Wu et al. (37)	CheckMate 078	2019	III	IIIB/IV/recurrence/progression	≥2L	Nivolumab (338)	Docetaxel (166)	60 (27–78)	78.8	29.8	39.7	60.3	40.7	50	9.3	13.5	86.3	OS

### Effects of PD-1 Inhibitors by Age Group

**Figure 3:**
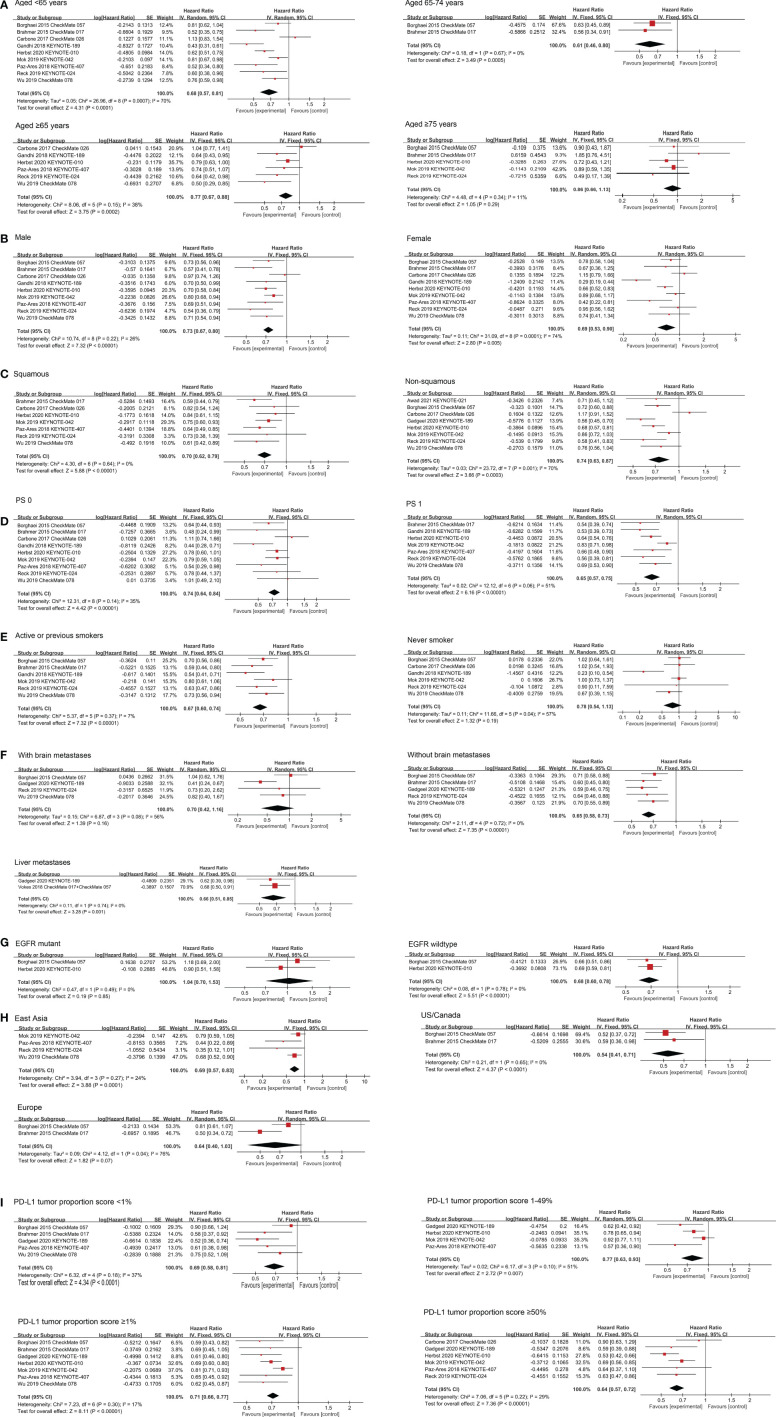
Forest plots of HRs comparing OS between PD-1 inhibitors based therapy and non-PD-1 inhibitor based therapy with respect to **(A)** age group, **(B)** gender, **(C)** histomorphological subtypes, **(D)** PS score, **(E)** smoking status, **(F)** metastases status/site, **(G)** EGFR mutation status, **(H)** region and **(I)** PD-L1 tumor proportion score.

Age group-specific survival data for NSCLC individuals was presented in nine publications. In individuals with age group <65 years (HR 0.68; 95% CI, 0.57–0.81; P <0.0001) and with age group ≥65 years (HR 0.77; 95% CI, 0.67–0.88; P = 0.0002), PD-1 inhibitors substantially increased OS relative to non-PD-1 inhibitor therapy. Interestingly, when the cutoff value of age group was set at 65–74 years and ≥75 years, we discovered OS benefit with the age group 65–74 years old individuals (HR 0.61; 95% CI, 0.46–0.80; P = 0.0005), while no OS benefit with the age group ≥75 years (HR 0.86; 95% CI, 0.66–1.13; P = 0.29) (**Figure 3A**). Subgroup analyses showed that in individuals with age group <65 years, pembrolizumab substantially enhanced OS not related with treatment line and treatment regimen, while nivolumab only improved OS in ≥2nd-line therapy (HR 0.72; 95% CI, 0.62–0.85; P = 0.0001). Nivolumab improved OS in individuals with age group 65–74 years in ≥2nd-line monotherapy (HR 0.61; 95% CI, 0.46–0.80; P = 0.0005) (**Table S1**). For PFS data from eight studies, PD-1 inhibitors substantially enhanced PFS compared with non-PD-1 inhibitor therapy in with age group <65 years (HR 0.71; 95% CI, 0.56–0.89; P = 0.003), and ≥65 years individuals (HR 0.76; 95% CI, 0.58–0.99; P = 0.04). Surprisingly, we did not observe PFS benefit in 65–74 years (HR 0.71; 95% CI, 0.40–1.28; P = 0.26), and ≥75 years individuals (HR 1.24; 95% CI, 0.73–2.11; P = 0.43) (**Figure S1A** and **Table S2**).

### Effects of PD-1 Inhibitors by Gender

Nine studies have examined the potency of PD-1 inhibitors in both gender individuals about OS. The comprehensive results showed that PD-1 inhibitors substantially enhanced OS in both gender NSCLC individuals compared with non-PD-1 inhibitor therapy (HR 0.73; 95% CI, 0.67–0.80; P <0.00001 for male; HR 0.69; 95% CI, 0.53–0.90; P = 0.0005 for female) (**Figure 3B**). Subgroup analyses showed that in male individuals, pembrolizumab substantially enhanced OS not related with treatment line and treatment regimen. Nivolumab substantially enhanced OS in ≥2nd-line therapy (HR 0.68; 95% CI, 0.57–0.80; P <0.00001) or monotherapy (HR 0.74; 95% CI, 0.60–0.91; P = 0.005). In female individuals, we found that pembrolizumab and nivolumab both improved OS in ≥2nd-line therapy (HR 0.66; 95% CI, 0.52–0.83; P = 0.0004 for pembrolizumab; HR 0.75; 95% CI, 0.59–0.96; P = 0.02 for nivolumab), but not in 1st-line therapy. Pembrolizumab enhanced OS in both monotherapy (HR 0.77; 95% CI, 0.85–0.91; P = 0.002) and combination therapy (HR 0.32; 95% CI, 0.23–0.46; P <0.00001), but not nivolumab in female individuals (**Table S1**). In the aspect of PFS data from eight studies, which substantially enhanced PFS in male (HR 0.69; 95% CI, 0.58–0.82; P <0.0001) but not in female individuals (HR 0.80; 95% CI, 0.59–1.10; P = 0.17) (**Figure S1B** and **Table S2**).

### Effects of PD-1 Inhibitors by Histomorphological Subtypes

The potency of PD-1 inhibitors on squamous and non-squamous NSCLC was studied in seven and eight studies, respectively. The integrated findings revealed that PD-1 inhibitors obviously enhanced OS in both squamous (HR 0.70; 95% CI, 0.62–0.79; P <0.00001) and non-squamous NSCLC (HR 0.74; 95% CI, 0.63–0.87; P = 0.0003) (**Figure 3C**). Subgroup analyses by the therapy line showed that in squamous NSCLC patients, pembrolizumab only benefits from 1st-line treatment (HR 0.71; 95% CI, 0.60–0.83; P <0.0001) and nivolumab only benefits from receiving ≥2nd-line treatment (HR 0.60; 95% CI, 0.47–0.75; P <0.0001). Subgroup analyses by the treatment regimen showed that pembrolizumab substantially enhanced OS from both monotherapy (HR 0.77; 95% CI, 0.65–0.92; P= 0.003) and combination therapy (HR 0.64; 95% CI, 0.49–0.85; P= 0.002), and nivolumab prolonged survival as monotherapy (HR 0.64; 95% CI, 0.53–0.79; P <0.0001). In non-squamous NSCLC individuals, pembrolizumab substantially enhanced OS not related with treatment line and treatment regimen, while nivolumab only improved OS in ≥2nd-line therapy (HR 0.73; 95% CI, 0.62–0.87; P = 0.0003) (**Table S1**). When it comes to PFS data from nine studies, which substantially enhanced PFS both in squamous NSCLC individuals (HR 0.63; 95% CI, 0.56–0.72; P <0.00001) and in non-squamous NSCLC individuals (HR 0.75; 95% CI, 0.58–0.99; P = 0.04) (**Figure S1C** and **Table S2**).

### Effects of PD-1 Inhibitors by ECOG PS Score

For individuals with PS 0, nine studies examined the effectiveness of PD-1 inhibitors, while for individuals with PS 1, seven studies investigated the effectiveness. The combined results showed that compared with non-PD-1 inhibitor treatment, both individuals with PS 0 (HR 0.74; 95% CI, 0.64–0.84; P <0.00001) and PS 1 (0.65 HR; 95% CI, 0.57–0.75; P <0.00001) realized OS enhancements after applying PD-1 inhibitors (**Figure 3D**). For patients with PS 0, subgroup analyses by the treatment line showed that pembrolizumab only benefits from 1st-line treatment (HR 0.67; 95% CI, 0.54–0.83; P = 0.0002) and nivolumab only benefits from ≥2nd-line treatment (HR 0.66; 95% CI, 0.49–0.89; P = 0.007). Subgroup analyses by the treatment regimen showed that pembrolizumab enhanced OS in both monotherapy (HR 0.78; 95% CI, 0.65–0.94; P = 0.008) and combination therapy (HR 0.48; 95% CI, 0.33–0.69; P = 0.0001), but not nivolumab. In patients with PS 1, pembrolizumab substantially enhanced OS not related with treatment line and treatment regimen, nivolumab improved OS as ≥2nd-line monotherapy (HR 0.62; 95% CI, 0.51–0.76; P <0.00001) (**Table S1**). For PFS data from eight studies, which substantially enhanced PFS in individuals with PS 1 (HR 0.65; 95% CI, 0.59–0.72; P <0.00001) but not in individuals with PS 0 (HR 0.76; 95% CI, 0.53–1.10; P = 0.15) (**Figure S1D** and **Table S2**).

### Effects of PD-1 Inhibitors by Smoking Status

PD-1 inhibitors were found to be more effective than non-PD-1 inhibitor medication in improving OS in individuals who were either actively smoking or had previously smoked six various studies (HR 0.67; 95% CI, 0.60–0.74; P <0.00001) (**Figure 3E**). Subgroup analyses by the treatment line showed that pembrolizumab benefits from 1st-line treatment (HR 0.65; 95% CI, 0.52–0.82; P = 0.0003) while nivolumab benefits from ≥2nd-line treatment (HR 0.68; 95% CI, 0.59–0.79; P <0.00001). Subgroup analyses by the treatment regimen showed that pembrolizumab improved OS in both monotherapy (HR 0.72; 95% CI, 0.59–0.88; P = 0.002) and combination therapy (HR 0.54; 95% CI, 0.41–0.71; P <0.0001), nivolumab benefits from monotherapy (HR 0.68; 95% CI, 0.59–0.79; P <0.00001) (**Table S1**). PD-1 inhibitors were found to be effective in six investigations in individuals who had never smoked. Cancer individuals who received PD-1 inhibitors and those who received conventional treatment had no statistically significant difference in survival (HR 0.78; 95% CI, 0.54–1.13; P = 0.19) (**Figure 3E**). An analysis of subgroups showed that only the combination therapy of pembrolizumab, pemetrexed and platinum was observed for survival benefit in individuals who never smoked (HR 0.23; 95% CI, 0.10–0.54; P = 0.0007) (**Table S1**). In terms of PFS data from six studies, PFS was substantially enhanced in individuals who are active or were former smokers (HR 0.67; 95% CI, 0.55–0.82; P = 0.0001) but not in individuals who never smoked (HR 1.06; 95% CI, 0.60–1.86; P = 0.85) (**Figure S1E** and **Table S2**).

### Effects of PD-1 Inhibitors by Metastatic Status/Site

In individuals with asymptomatic brain metastases, there were four studies reporting data on overall survival and progression-free survival. Individuals on PD-1 inhibitors-based therapy had an OS rate of 0.70, with a 95% CI of 0.42–1.16 (P = 0.16), but a prolonged progression-free survival rate of 0.57, with a 95% CI of 0.43–0.76 (P = 0.0001) (**Figure 3F** and **Figure S1F**). Subgroup analyses showed that 1st-line therapy based on pembrolizumab, had better OS (HR 0.44; 95% CI, 0.27–0.70; P = 0.0006) and PFS (HR 0.44; 95% CI, 0.29–0.67; P = 0.0001) than those who received non-PD-1 inhibitor treatment (**Tables S1**, **S2**).

Individuals without brain metastases were the focus of five studies that examined the OS. Individuals without brain metastases who received PD-1 inhibitors had a longer OS (HR 0.65; 95% CI, 0.58–0.73; P <0.00001) compared to those who received non-PD-1 inhibitor therapy (**Figure 3F**). Subgroup analyses by the treatment line showed that in individuals without brain metastases, pembrolizumab benefits from 1st-line treatment (HR 0.60; 95% CI, 0.50–0.73; P <0.00001) and nivolumab benefits from ≥2nd-line treatment (HR 0.68; 95% CI, 0.59–0.78; P <0.00001). Subgroup analyses by the treatment regimen showed that pembrolizumab substantially enhanced OS both monotherapy (HR 0.64; 95% CI, 0.46–0.88; P = 0.006) and combination therapy (HR 0.59; 95% CI, 0.46–0.75; P <0.0001), and nivolumab prolonged survival as monotherapy (HR 0.68; 95% CI, 0.59–0.78; P <0.00001) (**Table S1**). In terms of PFS, we also observed survival benefits in patients without brain metastases (HR 0.65; 95% CI, 0.50–0.85; P = 0.002) (**Figure S1F** and **Table S2**).

Individuals with liver metastases were followed up in three RCTs as part of two investigations. Cancer individuals with liver metastases who were given with PD-1 inhibitors had a longer OS (HR 0.66; 95% CI, 0.51–0.85; P = 0.001) compared to those who received non-PD-1 inhibitor therapy (**Figure 3F**). According to a single research, the combination of pembrolizumab with chemotherapy was found to be significantly more successful than non-PD-1 inhibitor therapy in 1st-line treatment (HR, 0.62; 95% CI, 0.39–0.98; P = 0.04). According to the combined results of the investigations CheckMate 057 and CheckMate 017, individuals with liver metastases who received nivolumab as a ≥2nd-line monotherapy (HR 0.68; 95% CI, 0.50–0.91; P = 0.01) had a longer OS than those who received docetaxel (**Table S1**).

### Effects of PD-1 Inhibitors by Driver Mutation Status

Results in terms of OS were published in two studies, both of which assessed whether PD-1 inhibitor monotherapy in the ≥2nd line was superior to docetaxel in individuals with EGFR mutations. Combined results showed that PD-1 inhibitors provided longer OS for EGFR wild-type individuals (HR 0.68; 95% CI, 0.60–0.78; P <0.00001), while did not for EGFR mutant individuals (HR 1.04; 95% CI, 0.70–1.53; P = 0.85) compared with non-PD-1 inhibitor therapy (**Figure 3G**). In terms of PFS, we did not observe PFS benefit in EGFR mutation-positive individuals (HR 1.10; 95% CI, 0.50–2.42; P = 0.81), or EGFR wild-type individuals (HR 0.69; 95% CI, 0.48–0.99; P = 0.05) (**Figure S1G** and **Table S2**).

### Effects of PD-1 Inhibitors by Region

In individuals from East Asia, the effectiveness of PD-1 inhibitors has been demonstrated in four clinical trials. PD-1 inhibitors therapy was found to substantially improve OS when compared to non-PD-1 inhibitor therapy (HR 0.69; 95% CI, 0.57–0.83; P = 0.0001), according to the combined data (**Figure 3H**). Subgroup analyses showed that pembrolizumab substantially enhanced OS in 1st-line therapy (HR 0.69; 95% CI, 0.54–0.90; P = 0.005) (**Table S1**). Two studies reported the potency of PD-1 inhibitors in European individuals, and showed that nivolumab monotherapy as ≥2nd-line therapy did not prolong OS compared to non-PD-1 inhibitor treatment (HR 0.64; 95% CI, 0.40–1.03; P = 0.07) (**Figure 3H** and **Table S1**). Two studies reported the potency of PD-1 inhibitors in U.S./Canadian individuals, and showed that nivolumab monotherapy as ≥2nd-line therapy provided longer OS than non-PD-1 inhibitor treatment (HR 0.54; 95% CI, 0.41–0.71; P <0.0001) (**Figure 3H** and **Table S1**). In terms of PFS, similar to OS results, PD-1 inhibitors improved PFS in East Asian (HR 0.46; 95% CI, 0.29–0.71; P = 0.0006), U.S./Canadian (HR 0.65; 95% CI, 0.50–0.84; P = 0.001) populations compared to non-PD-1 inhibitor, but did not prolong survival in Europeans (HR 0.78; 95% CI, 0.43–1.39; P = 0.39) (**Figure S1H** and **Table S2**).

### Effects of PD-1 Inhibitors by PD-L1 Tumor Proportion Score

There was five researches that looked at the potency of PD-1 inhibitors in individuals with PD-L1 TPS <1%, and the combined results showed that PD-1 inhibitors therapy substantially enhanced OS when compared to non-PD-1 inhibitor therapy (HR 0.69; 95% CI, 0.58–0.81; P <0.0001) (**Figure 3I**). Subgroup analyses by the treatment line showed that pembrolizumab benefits from 1st-line treatment (HR 0.55; 95% CI, 0.41–0.73; P <0.0001) and nivolumab benefits from ≥2nd-line treatment (HR 0.77; 95% CI, 0.63–0.96; P = 0.02). Subgroup analyses by the treatment regimen showed that pembrolizumab substantially enhanced OS as combination therapy (HR 0.55; 95% CI, 0.41–0.73; P <0.0001), and nivolumab prolonged survival as monotherapy (HR 0.77; 95% CI, 0.63–0.96; P = 0.02) (**Table S1**).

Seven studies reported the potency of PD-1 inhibitors in individuals with TPS ≥1%. The aggregated findings indicated that PD-1 inhibitors therapy prolonged OS (HR 0.71; 95% CI, 0.66–0.77; P <0.00001) (**Figure 3I**). Subgroup analyses showed that pembrolizumab substantially enhanced OS not related with treatment line and treatment regimen, and only observed benefit in ≥2nd-line monotherapy based on nivolumab (HR 0.63; 95% CI, 0.51–0.77; P <0.00001) (**Table S1**).

In the four trials that looked at the effectiveness of PD-1 inhibitors in individuals with TPS 1–49%, it was discovered that treatment with the medicine significantly enhanced OS when compared to treatment without the drug (HR 0.77; 95% CI, 0.63–0.93; P = 0.007) (**Figure 3I**). Analysis of subgroups by the treatment line showed that receiving ≥2nd-line treatment based on pembrolizumab prolonged OS (HR 0.78; 95% CI, 0.65–0.94; P = 0.009), but not in 1st-line treatment (HR 0.72; 95% CI, 0.52–1.01; P = 0.06). Pembrolizumab improved OS as both monotherapy (HR 0.85; 95% CI, 0.75–0.97; P = 0.01) and combination therapy (HR 0.60; 95% CI, 0.44–0.81; P = 0.0007) (**Table S1**).

According to the cumulative findings from six studies, PD-1 inhibitors therapy markedly enhanced OS over non-PD-1 inhibitor therapy in individuals with TPS ≥50% (HR 0.64; 95% CI, 0.57–0.72; P <0.00001) (**Figure 3I**). Subgroup analyses showed pembrolizumab substantially enhanced OS not related with treatment line and treatment regimen (**Table S1**).

A total of ten studies reported PFS data of NSCLC individuals stratified by PD-L1 tumor proportion score, and were found to benefit from PD-1 inhibitors compared to non-PD-1 inhibitor therapy with PD-L1 TPS <1% (HR 0.74; 95% CI, 0.58–0.95; P = 0.02), TPS ≥1% (HR 0.60; 95% CI, 0.42–0.86; P = 0.005), and TPS ≥50% (HR 0.58; 95% CI, 0.43–0.79; P = 0.0006), respectively, while PFS benefit was not observed in individuals with TPS 1–49% (HR 0.68; 95% CI, 0.41–1.12; P = 0.13) (**Figure S1I** and **Table S2**).

### Drug Selection

The clinical and molecular features could be used to predict the efficacy of pembrolizumab and nivolumab in different treatment lines and treatment regimens, as shown in **Table 2** and **Table S3**.

According to the cumulative findings from our results, PD-1 inhibitor therapy markedly enhanced OS over non-PD-1 inhibitor therapy in 1st-line and ≥2nd-line treatment in patients with different characteristics. Analysis of subgroups showed that in 1st-line treatment, pembrolizumab monotherapy and combination therapy substantially enhanced OS compared to non-PD-1 inhibitor treatment. In ≥2nd-line treatment, monotherapy based on pembrolizumab and nivolumab substantially prolonged patients OS.

**Table 2:** Different treatment lines and regimens with OS benefited from PD-1 inhibitor over non-PD-1 inhibitors in targeted patients.

Line	Regimen	Population	No. of studies	HR	95% CI	P-value
1st Line	P monotherapy	Aged <65 years	2	0.78	0.65–0.93	0.005
		Aged ≥65 years	1	0.64	0.42–0.98	0.04
		Squamous	2	0.74	0.61–0.92	0.005
		Active or previous smoker	2	0.72	0.59–0.88	0.002
		Without brain metastases	1	0.64	0.46–0.88	0.006
		PD-L1 TPS ≥1%	1	0.81	0.71–0.93	0.003
		PD-L1 TPS ≥50%	2	0.67	0.57–0.80	<0.00001
	P combined therapy	Aged <65 years	2	0.47	036–0.61	<0.00001
		Aged ≥65 years	2	0.69	0.53–0.91	0.007
		Male	2	0.70	0.56–0.88	0.002
		Female	2	0.32	0.23–0.46	<0.00001
		Squamous	1	0.64	0.49–0.85	0.002
		Non-squamous	2	0.59	0.48–0.72	<0.00001
		PS 0	2	0.48	0.33–0.69	0.0001
		PS 1	2	0.59	0.47–0.74	<0.00001
		Active or previous smoker	1	0.54	0.41–0.71	<0.0001
		Never smoker	1	0.23	0.10–0.54	0.0007
		With brain metastases	1	0.41	0.24–0.67	0.0005
		Without brain metastases	1	0.59	0.46–0.75	<0.0001
		Liver metastases	1	0.62	0.39–0.98	0.04
		East Asia	1	0.44	0.22–0.89	0.02
		PD-L1 TPS <1%	2	0.55	0.41–0.73	<0.0001
		PD-L1 TPS ≥1%	2	0.62	0.50–0.77	<0.0001
		PD-L1 TPS 1–49%	2	0.60	0.44–0.81	0.0007
		PD-L1 TPS ≥50%	2	0.60	0.44–0.84	0.002
	N monotherapy	None				
	N combined therapy	None				
≥2nd Line	P monotherapy	Aged <65 years	1	0.62	0.51–0.75	<0.00001
		Male	1	0.70	0.58–0.84	0.0001
		Female	1	0.66	0.52–0.83	0.0004
		Non-squamous	1	0.68	0.57–0.81	<0.0001
		PS 1	1	0.64	0.54–0.76	<0.00001
		EGFR mutant	1	0.69	0.59–0.81	<0.00001
		PD-L1 TPS ≥1%	1	0.69	0.60–0.80	<0.00001
		PD-L1 TPS 1–49%	1	0.78	0.65–0.94	0.009
		PD-L1 TPS ≥50%	1	0.53	0.42–0.66	<0.00001
	P combined therapy	None				
	N monotherapy	Aged <65 years	3	0.72	0.62–0.85	0.0001
		Aged ≥65 years	1	0.50	0.29–0.85	0.01
		Aged 65–74 years	2	0.61	0.46–0.80	0.0005
		Male	3	0.68	0.57–0.80	<0.00001
		Female	3	0.75	0.59–0.96	0.02
		Squamous	2	0.60	0.47–0.75	<0.0001
		Non-squamous	2	0.73	0.62–0.87	0.0003
		PS 0	3	0.66	0.49–0.89	0.007
		PS 1	2	0.62	0.51–0.76	<0.00001
		Active or previous smoker	3	0.68	0.59–0.79	<0.00001
		Without brain metastases	3	0.68	0.59–0.78	<0.00001
		Liver metastases	1	0.68	0.50–0.91	0.01
		EGFR mutant	1	0.66	0.51–0.86	0.002
		East Asia	1	0.68	0.52–0.90	0.007
		US/Canada	2	0.54	0.41–0.71	<0.0001
		PD-L1 TPS <1%	3	0.77	0.63–0.96	0.02
		PD-L1 TPS ≥1%	3	0.63	0.51–0.77	<0.00001
	N combined therapy	None				

### Sensitivity Analysis and Publication Bias

The two trials of KEYNOTE-021 and PROLUNG included a small number of individuals, thus the sensitivity analysis was carried out by excluding these two trials. The findings indicated that the predictive value of numerous clinical and molecular PD-1 inhibitors in the treatment of OS remained steady during the course of the analysis. Furthermore, we excluded the CheckMate 078 trial, which only provided HR, 95% CI was estimated from the forest plot, and found that the conclusion of the preliminary analysis had not changed. Besides, we found no significant publication bias according to the OS and PFS funnel of each subgroup (**Figures S2**, **S3**).

## Discussion

In earlier meta-analyses of the effects of PD-1 inhibitors on NSCLC clinical and molecular characteristics, a smaller number of individuals were included than in the active study. Eleven RCTs involving 5,887 patients with advanced NSCLC made up our meta-analysis. Using the most recent clinical data, we seek to determine whether there are useful and cost-effective clinical and molecular pathological markers that can be used to predict the potency of PD-1 inhibitor therapy and guide treatment options for people who may benefit from pembrolizumab or nivolumab in the field.

Based on previous clinical trials, it remains controversial whether PD-1 inhibitors benefit elderly patients with NSCLC (39–43). When treating cancer in the elderly, it is not apparent if pembrolizumab or nivolumab should be utilized. In our meta-analysis, we found that treatment with PD-1 inhibitors in individuals <65 years old is more likely to get OS benefit from pembrolizumab. In individuals with 65–74 years, the OS benefit only was observed from ≥2nd-line monotherapy based on nivolumab, but the therapeutic effect of pembrolizumab needs to be further clarified. Furthermore, we did not find treatment benefit for PD-1 inhibitors in patient from age group 65–74 years for PFS; this may be the reason for the heterogeneity of the experiment or the small scale of this population. In individuals ≥ 75 years old, our result had not shown that PD-1 inhibitors are far more powerful than non-PD-1 inhibitor therapy; this may be due to, firstly, they are more likely to have a poor PS when they have comorbidities, which indicates that they will gain less benefit from medicine (44, 45). Secondly, elderly people are associated with a functional decline of the immune system called immune-senescence so that they are unable to restore anti-tumor activity (46), and thirdly, older adults experience more frequent or severe toxicities from immunotherapy, and they may be more vulnerable to treatment-related toxicities (47). Individuals from the age group of ≥75 make about half of all those diagnosed with NSCLC, and that figure is only going to rise. Additionally, a cost–benefit analysis should be performed (48, 49). Thus, we need to be cautious about using PD-1 inhibitors and there is a need for more explorations of PD-1 inhibitors in NSCLC individuals aged ≥75 years. The meta-analysis by Elias et al. explored the potency of PD-1/PD-L1 inhibitors in multiple kinds of solid tumor of the elderly population and found that this benefit was consistent in the subgroups of individuals with age groups <65 and ≥65, with HR of 0.68 (95% CI, 0.61–0.75) and HR of 0.64 (95% CI, 0.54–0.76). In the subgroup analyses of four PD-1 inhibitor treatment trials, they did not observe the improvement of OS in elderly individuals aged ≥75 years (HR Value 0.86; 95% CI, 0.41–1.83) (41). Zhang et al. conducted a meta-analysis of the potency of PD-1/CTLA-4 inhibitors in elderly individuals with lung cancer and showed that immunotherapy failed to prove that individuals ≥75 years old were statistically beneficial (HR Value 0.90; 95% CI, 0.64 to 1.25) (43). The outcomes of these studies provided additional support for our meta-analysis. Following the meta-analysis of Wu et al., individuals with age group 65 and older had considerably greater overall survival with PD-1 inhibitors than those receiving chemotherapy; however individuals with age group 75 and older had significantly shorter overall survival with PD-1 inhibitors. They discovered, however, that nivolumab was related with a superior overall survival (OS) and progression-free survival (PFS) in individuals with age group 65 and older with NSCLC (42). In our meta-analysis, we found that pembrolizumab was significantly associated with better OS (HR Value 0.73; 95% CI, 0.62–0.86; P-value = 0.0002) and PFS (HR Value 0.69; 95% CI, 0.52–0.91; P-value = 0.008) in with age group ≥65 years old individuals, while nivolumab did not substantially enhanced OS and PFS. The reason why our results varied from that of Wu et al. is that we included more RCTs and more individuals.

The variable of gender is well-known, and it has an impact on both innate and adaptive immune responses (50). The effect of the gender of individuals on the potency of PD-1 inhibitors as treatment in NSCLC still remains controversial (51–55). Our meta-analysis showed that for male individuals given with PD-1 inhibitors, OS and PFS were improved compared to those given with non-PD-1 inhibitor, whereas in females, we found only benefited in OS but not in PFS. Previous study also suggested that male was a favorable prognostic factor for PFS and male benefits more than female, although the OS for both genders can be improved by PD-1 inhibitors (52, 55, 56). Differences between men and women may be explained by the fact that women have a stronger immune environment in their bodies, which leads to more effective cancer cell escape mechanisms (due to stronger innate and adaptive immune responses), which may make PD-1 inhibitors less effective in women than in men and may lead to increased resistance against PD-1 inhibitors (50, 57, 58). On the other hand, men with higher smoking frequency associated with high TMB (59, 60), whereby further genetic mutations generate neoantigens, reflecting the high antigenicity of tumors (61, 62), may obtain greater benefit from PD-1 inhibitors in males. While common driver mutations in lung adenocarcinoma associated with low-TMB, usually female patients get higher EGFR mutations rate probability to have lower TMB (63) that lead females not respond well to immunotherapy. Consequently, improving the immune environment and the antigenicity of tumor in female patients may be a useful strategy, which is worth exploring in the future. Immunotherapy research and development should take gender disparities in immune response into consideration. We should also consider gender differences when we seek biomarkers which predict immunotherapy response.

Based on our analysis of subgroups of patients with histomorphological subtypes in NSCLC, we recommend individuals with squamous cell carcinoma to consider pembrolizumab as 1st-line treatment and nivolumab as ≥2nd-line treatment. We recommend pembrolizumab therapy as 1st-line treatment and pembrolizumab or nivolumab as ≥2nd-line treatment for individuals with non-squamous cell carcinoma. Both squamous and non-squamous cell carcinoma individuals can benefit from pembrolizumab monotherapy and combination therapy. Furthermore, the combination therapy of nivolumab in patients with any histomorphological subtypes remains to be explored.

The introduction of ICIs has substantially enhanced the prognosis of individuals with NSCLC, but only in individuals with ECOG PS of 0 or 1 (64). In our meta-analysis, both of the PS 0 and 1 patients given with PD-1 inhibitors achieved OS benefit compared with those given with non-PD-1 inhibitor. However, only PS 1 patients but not PS 0 patients acquired PFS benefit. When we performed a sensitivity analysis on patients with PS 0, excluding CheckMate 026, the 1st-line monotherapy based on nivolumab, and we unexpectedly observed a significant PFS benefit after applying PD-1 inhibitors. Therefore, the ECOG PS 0, 1 seems not an appropriate predictor for evaluating the potency of PD-1 inhibitors.

In addition, the potency of PD-1 inhibitors in various smoking status was also analyzed, and found that a survival benefit of PD-1 inhibitors was observed in active or former smokers, but not in never smokers from our results. Some studies have shown that in NSCLC, smokers have a favorable trend of PD-1 inhibitor treatment compared with non-smokers (65–67) and support the result of our meta-analysis. This may be because smoking is considered to increase the mutation load in tumors and increase the expression of carcinogenic new antigens, thus activating an effective anti-tumor immune response (68). A combination strategy, rather than a single medicine, pembrolizumab, may be given more consideration in patients who have never smoked.

At present, PD-1 inhibitors have become an important treatment choice for individuals with distant metastasis (such as brain or liver metastasis). Our meta-analysis of a longer PFS but not OS for PD-1 inhibitors in patients with asymptomatic brain metastases does not support previous studies that patients with advanced brain metastasis of NSCLC given with PD-1/PD-L1 inhibitors obtained longer OS (69). Reasons for these conflicting findings include that these results are mixed with the PD-1/PD-L1 inhibitors, while our results specifically focused on the effects of the PD-1 inhibitors on individuals with asymptomatic brain metastases. In addition, the possibility that the unknown consequence of crossover at disease progression, and a significant heterogeneity existed in the results (P-value = 0.08; I² = 56%), which also may be the reason why PFS does not translate to OS improvement. Notably, although OS was not a statistically significant benefit in individuals given with PD-1 inhibitors, compared with non-PD-1 inhibitor treatment, we observed that 1st-line treatment based on pembrolizumab prolonged survival in brain metastases patients for both OS and PFS. Following a retrospective cohort study revealed the potential benefits of the use of pembrolizumab for patients with brain metastases (70). As for individuals without brain metastasis, both OS and PFS were improved after PD-1 inhibitor treatment. Individuals with liver metastases had OS benefit from PD-1 inhibitors, and may benefit from 1st-line combined treatment of pembrolizumab with chemotherapy and ≥2nd-line nivolumab monotherapy from our subgroup analysis. Consequently, metastatic status/site may be independent predictors of survival outcome in NSCLC individuals given with PD-1 inhibitors.

The connection between PD-1 inhibitors and driving mutations has long been a focus of investigation. In this investigation, we discovered that EGFR mutation status was linked to the potency of PD-1 inhibitors. Individuals with EGFR wild-type cells benefited from PD-1 inhibitors, whereas those with EGFR mutations did not. On EGFR mutant NSCLC, it is possible that PD-1 inhibitors are ineffective due to the fact that: NSCLC individuals with EGFR wild-type and high levels of PD-L1 expression may benefit better from immune checkpoint blockade therapy than standard chemotherapy (71–73). Additionally, TMB levels in individuals with EGFR mutant tumors were shown to be lower than those in individuals with EGFR wild-type tumors, suggesting that TMB may be a contributing factor to the poor potency of immunomodulatory drugs in these individuals (74–77). Numerous studies have demonstrated that a high CD8^+^ T infiltration rate is related with a favorable prognosis for NSCLC (78–80). Nonetheless, tumors harboring EGFR mutations frequently have a reduced percentage group of CD8^+^ tumor-infiltrating lymphocytes (TILs) (77, 81), which may result in immunological dysfunction and a poor prognosis (82). Additionally, CD73 expression is increased in the tumor cells of NSCLC individuals with EGFR mutations (83). High expression of CD73 can cause immunosuppression *via* decreased T-cell activation and effector function, and hence reduced benefit from checkpoint inhibitor therapies (84, 85). Natural killer (NK) cells, CD4^+^ T cells, and CD8^+^ T cells are all found in high numbers in tumors with EGFR mutations (86). Tregs secrete interleukin-10, -35, and transforming growth factor− (TGF−) in order to suppress the anti-tumor immune response mediated by NK cells, CD4^+^ T cells, and CD8^+^ T cells (87).

Actively, PD-1 inhibitors are widely used in clinical practice, and this region information will be essential to bring maximum benefit to individuals with NSCLC. From our meta-analysis, different regions could predict clinical potency of PD-1 inhibitors, where survival benefit of PD-1 inhibitors was observed in individuals from East Asia and the U.S./Canada, but not in European individuals. Given the analysis of subgroups, due to the small number of individuals analyzed, the results should be interpreted with caution. Individuals with advanced NSCLC of different regions have different clinical, genetic characteristics, and socioenvironmental make-up that may influence their response to PD-1 inhibitors (88). It is possible that there is some yet unknown mechanism that could explain the differences, or it is far more likely that this statistical significance is due to chance (89). Therefore, further research and further confirmatory studies were required with large numbers of patients applying PD-1 inhibitors in different regions.

In our meta-analysis, we found that PD-1 inhibitor treatment substantially enhanced OS compared with non-PD-1 inhibitor therapy in individuals with any level of PD-L1 expression. Subgroup analyses showed that both pembrolizumab and nivolumab substantially enhanced OS of individuals with PD-L1 TPS <1% and TPS ≥1%, and pembrolizumab significantly prolonged OS of individuals with PD-L1 TPS ≥50%. We did not found survival benefit in individuals with TPS 1–49% given with 1st-line based on pembrolizumab, but when we performed a sensitivity analysis and excluded Keynote-042 (1st monotherapy based on pembrolizumab), the results were statistically significant when compared with non-PD-1 inhibitor treatment, that pembrolizumab combination therapy as 1st-line therapy substantially enhanced OS in individuals with PD-L1 TPS 1–49% (HR Value 0.60; 95% CI, 0.44–0.81; P-value = 0.0007). A meta-analysis had shown similar results that pembrolizumab combination therapy seem to be reasonable 1st-line regimens when PD-L1 TPS 1–49% (HR Value 0.55; 95% CI, 0.34–0.89; P-value = 0.015); by contrast, there was no significant statistical difference in ICI monotherapy as 1st-line therapy (90). Secondly, pembrolizumab combination therapy shows the advantage groups of early disease control in improving PFS and preventing early disease progression in individuals with PD-L1 TPS 1–49% (90, 91).

Despite the fact that our research generated helpful insights, we recognize that it has several limits. To begin, our analysis found publication bias and heterogeneity, which might be accounted by differences in the characteristics of the research that was included in the study. We found that heterogeneity among the selected investigations has a small impact on our principal conclusions, as evidenced by our subgroup analyses and sensitivity analysis results. Second, the data were extracted from summary data rather than from the individuals from each trial, which might lead to heterogeneity among the various studies. Third, because our study was based on correlations rather than causal findings, further investigation is needed to understand the mechanisms by which various clinical and molecular characteristics can predict PD-1 inhibitor potency, and to determine whether other biomarkers have a relationship with PD-1 inhibitor potency. Fourth, rather than research exploring the effect of specific clinicopathological characteristics on the effectiveness of PD-1 immune checkpoint blocking medicine, our meta-analysis is based on the results of planned subgroup analyses of published randomized controlled trials. Various clinicopathological characteristics such as smoking status and squamous cell carcinoma may be associated with one another. When we focus primarily on a single trait, it is possible that other confounding variables have an effect on the survival outcomes. Fifth, because not all results showed all subgroup characteristics, the effects of those that did were omitted in the analysis of subgroups, which may have resulted in imprecise categorization of factors leading to heterogeneity.

From our meta-analysis, in patients with NSCLC, age group, smoking status, metastasis status/site, EGFR mutation status, and region can predict the potency of PD-1 inhibitors, which individuals with age group <65 years, 65–74 years, active or previous smokers, without brain metastasis, liver metastasis, EGFR wild-type, East Asia and U.S./Canada may benefit from PD-1 inhibitor treatment. PD-1 inhibitors can improve OS regardless of gender, histomorphological subtypes, ECOG PS, and PD-L1 TPS. Patients with age group <65 years old, male, non squamous cell carcinoma, PS 1, TPS ≥1%, and TPS ≥50% benefited from pembrolizumab treatment not related with treatment line and treatment regimen.

In the treatment of NSCLC, the relationship between gene expression and the potency of chemotherapy is not intimate. Targeted therapy is an intervention that targets specific genes of a patient. Our meta-analysis showed that the efficacy of PD-1 inhibitors may be associated with clinical and molecular features, which maybe represent the genomic “terrain map” of patients. The so-called “terrain map” of genome is the specific picture of gene expression in patients with certain particular clinical and molecular characteristics, which may be related to the immune anti-tumor and tumor immune microenvironment. Therefore, the exploration of the overview of the genomic “terrain map” of patients is expected to comprehensively and deeply understand the relationship between different clinical and molecular characteristics and the efficacy of PD-1 inhibitors, so as to achieve the purpose of individualized therapy, which is not for a specific individual, but for a group of patients with the similar certain clinical and molecular characteristics, with the specific genetic “terrain map”.

In conclusion, specific clinical characteristics can be used to predict the potency of PD-1 inhibitors. They are useful in the practical application of PD-1 inhibitors to better guide the treatment of NSCLC patients and to acquire more accurate NSCLC treatment in immunotherapy. Additionally, our article may aid in the identification of patients for PD-1 inhibitor therapy and may serve as a reference for the design of future clinical trials. Subgroup analyses suggest that when selecting PD-1 inhibitor therapy for pembrolizumab and nivolumab, careful consideration should be given to the appropriate population, in order to achieve the precise and individualized treatment purpose of immunotherapy.

## Data Availability Statement

The original contributions presented in the study are included in the article/**Supplementary Material**, further inquiries can be directed to the corresponding author.

## Author Contributions

Conception and design: PC, GWH, WJL. Collection and assembly of data: GWH, WJL. Assessed the eligibilities of feasible studies: WJL, GWH. Statistical analysis: GWH, WJL. Methodology and visualization: GWH. Wrote the first draft of the manuscript: GWH, WJL. Revised and edited themanuscript: GWH. Final approval of manuscript: all authors.

## Funding

This work was funded by the Tianjin Major Disease Prevention and Control Science and Technology project, Tianjin Municipal Science and Technology Bureau (18ZXDBSY00050).

## Conflict of Interest

The authors declare that the research was conducted in the absence of any commercial or financial relationships that could be construed as a potential conflict of interest.

## Publisher’s Note

All claims expressed in this article are solely those of the authors and do not necessarily represent those of their affiliated organizations, or those of the publisher, the editors and the reviewers. Any product that may be evaluated in this article, or claim that may be made by its manufacturer, is not guaranteed or endorsed by the publisher.
