# Supplementary material for: Inhibitors of PD-1 in Non-Small Cell Lung Cancer: A Meta-Analysis of Clinical and Molecular Features
Source: Front Immunol. 2022 Apr 5;13:875093. doi: 10.3389/fimmu.2022.875093 (PMC9037098; doi:10.3389/fimmu.2022.875093)
Supplement: Supplementary file 1 [file DataSheet_1.pdf]

Table S1 Analyses of OS in subgroups of patients with varying clinical and molecular features

Population	Subgroup	No. of studies	Test of association			Test of heterogeneity	
			HR	95%CI	P value	I^2	P value
Aged <65 years	Total	9	0.68	0.57-0.81	<0.0001	70%	0.0007
	1st Line	5	0.67	0.48-0.94	0.02	81%	0.0003
	Nivolumab	1	1.13	0.83-1.54	0.44		
	Pembrolizumab	4	0.59	0.42-0.82	0.002	74%	0.009
	≥2nd Line	4	0.68	0.60-0.77	<0.00001	45%	0.14
	Nivolumab	3	0.72	0.62-0.85	0.0001	49%	0.14
	Pembrolizumab	1	0.62	0.51-0.75	<0.00001		
	monotherapy	7	0.74	0.63-0.88	0.0004	63%	0.01
	Nivolumab	4	0.78	0.60-1.03	0.08	70%	0.02
	Pembrolizumab	3	0.69	0.56-0.85	0.0005	53%	0.12
	combination therapy	2	0.47	0.36-0.61	<0.00001	0%	0.51
	Pembrolizumab	2	0.47	0.36-0.61	<0.00001	0%	0.51
	Nivolumab	4	0.78	0.60-1.03	0.08	70%	0.02
	Pembrolizumab	5	0.60	0.48-0.76	<0.0001	67%	0.02
Aged ≥65 years	Total	6	0.77	0.67-0.88	0.0002	38%	0.15
	1st Line	4	0.79	0.66-0.95	0.01	44%	0.15
	Nivolumab	1	1.04	0.77-1.41	0.79		
	Pembrolizumab	3	0.68	0.54-0.85	0.0008	0%	0.84
	≥2nd Line	2	0.67	0.43-1.04	0.07	59%	0.12
	Nivolumab	1	0.50	0.29-0.85	0.01		
	Pembrolizumab	1	0.79	0.63-1.00	0.05		
	monotherapy	4	0.76	0.58-0.99	0.04	57%	0.07
	Nivolumab	2	0.75	0.36-1.53	0.42	82%	0.02
	Pembrolizumab	2	0.76	0.62-0.93	0.007	0%	0.39
	combination therapy	2	0.69	0.53-0.91	0.007	0%	0.60
	Pembrolizumab	2	0.69	0.53-0.91	0.007	0%	0.60
	Nivolumab	2	0.75	0.36-1.53	0.42	82%	0.02
	Pembrolizumab	4	0.73	0.62-0.86	0.0002	0%	0.73
Aged 65-74 years	Total	2	0.61	0.46-0.80	0.0005	0%	0.67
	1st Line	0					
	≥2nd Line	2	0.61	0.46-0.80	0.0005	0%	0.67
	Nivolumab	2	0.61	0.46-0.80	0.0005	0%	0.67
	Pembrolizumab	0					
	monotherapy	2	0.61	0.46-0.80	0.0005	0%	0.67
	Nivolumab	2	0.61	0.46-0.80	0.0005	0%	0.67
	combination therapy	0					
	Nivolumab	2	0.61	0.46-0.80	0.0005	0%	0.67
	Pembrolizumab	0					
Aged ≥75 years	Total	5	0.86	0.66-1.13	0.29	11%	0.34
	1st Line	2	0.82	0.56-1.21	0.32	10%	0.29
	Pembrolizumab	2	0.82	0.56-1.21	0.32	10%	0.29
	≥2nd Line	3	0.91	0.62-1.33	0.62	38%	0.20
	Nivolumab	2	1.20	0.68-2.12	0.52	34%	0.22
	Pembrolizumab	1	0.72	0.43-1.21	0.21		
	monotherapy	5	0.86	0.66-1.13	0.29	11%	0.34

	Nivolumab	2	1.20	0.68-2.12	0.52	34%	0.22
	Pembrolizumab	3	0.78	0.58-1.07	0.12	0%	0.53
	combination therapy	0					
	Nivolumab	2	1.20	0.68-2.12	0.52	34%	0.22
	Pembrolizumab	3	0.78	0.58-1.07	0.12	0%	0.53
Male	Total	9	0.73	0.67-0.80	<0.00001	26%	0.22
	1st Line	5	0.77	0.69-0.87	<0.00001	44%	0.13
	Nivolumab	1	0.97	0.74-1.26	0.80		
	Pembrolizumab	4	0.74	0.65-0.83	<0.00001	21%	0.28
	≥2nd Line	4	0.69	0.61-0.78	<0.00001	0%	0.63
	Nivolumab	3	0.68	0.57-0.80	<0.00001	0%	0.44
	Pembrolizumab	1	0.70	0.58-0.84	0.0001		
	monotherapy	7	0.74	0.67-0.81	<0.00001	43%	0.10
	Nivolumab	4	0.74	0.60-0.91	0.005	54%	0.09
	Pembrolizumab	3	0.73	0.65-0.82	<0.00001	49%	0.14
	combination therapy	2	0.70	0.56-0.88	0.002	0%	0.95
	Pembrolizumab	2	0.70	0.56-0.88	0.002	0%	0.95
	Nivolumab	4	0.74	0.60-0.91	0.005	54%	0.09
	Pembrolizumab	5	0.72	0.65-0.80	<0.00001	0%	0.40
Female	Total	9	0.69	0.53-0.90	0.005	74%	0.0001
	1st Line	5	0.66	0.40-1.11	0.12	87%	<0.00001
	Nivolumab	1	1.15	0.79-1.66	0.47		
	Pembrolizumab	4	0.57	0.31-1.06	0.08	87%	<0.0001
	≥2nd Line	4	0.70	0.59-0.83	<0.0001	0%	0.84
	Nivolumab	3	0.75	0.59-0.96	0.02	0%	0.91
	Pembrolizumab	1	0.66	0.52-0.83	0.0004		
	monotherapy	7	0.80	0.70-0.91	0.0008	23%	0.26
	Nivolumab	4	0.85	0.70-1.04	0.12	17%	0.31
	Pembrolizumab	3	0.77	0.85-0.91	0.002	43%	0.17
	combination therapy	2	0.32	0.23-0.46	<0.00001	0%	0.34
	Pembrolizumab	2	0.32	0.23-0.46	<0.00001	0%	0.34
	Nivolumab	4	0.85	0.70-1.04	0.12	17%	0.31
	Pembrolizumab	5	0.60	0.40-0.89	0.01	83%	0.0001
Squamous	Total	7	0.70	0.62-0.79	<0.00001	0%	0.64
	1st Line	4	0.72	0.62-0.84	<0.0001	0%	0.77
	Nivolumab	1	0.82	0.54-1.24	0.34		
	Pembrolizumab	3	0.71	0.60-0.83	<0.0001	0%	0.71
	≥2nd Line	3	0.67	0.56-0.81	<0.0001	30%	0.24
	Nivolumab	2	0.60	0.47-0.75	<0.0001	0%	0.88
	Pembrolizumab	1	0.84	0.61-1.15	0.27		
	monotherapy	6	0.71	0.63-0.81	<0.00001	0%	0.57
	Nivolumab	3	0.64	0.53-0.79	<0.0001	0%	0.43
	Pembrolizumab	3	0.77	0.65-0.92	0.003	0%	0.83
	combination therapy	1	0.64	0.49-0.85	0.002		
	Pembrolizumab	1	0.64	0.49-0.85	0.002		
	Nivolumab	3	0.64	0.53-0.79	<0.0001	0%	0.43
	Pembrolizumab	4	0.73	0.63-0.85	<0.0001	0%	0.67
Non-squamous	Total	8	0.74	0.63-0.87	0.0003	70%	0.001
	1st Line	5	0.76	0.57-1.00	0.05	82%	0.0002

	Nivolumab	1	1.17	0.91-1.52	0.23		
	Pembrolizumab	4	0.68	0.52-0.87	0.002	70%	0.02
	≥2nd Line	3	0.71	0.63-0.80	<0.00001	0%	0.78
	Nivolumab	2	0.73	0.62-0.87	0.0003	0%	0.78
	Pembrolizumab	1	0.68	0.57-0.81	<0.0001		
	monotherapy	6	0.78	0.66-0.93	0.005	69%	0.006
	Nivolumab	3	0.86	0.63-1.18	0.35	78%	0.01
	Pembrolizumab	3	0.72	0.59-0.89	0.002	63%	0.07
	combination therapy	2	0.59	0.48-0.72	<0.00001	0%	0.36
	Pembrolizumab	2	0.59	0.48-0.72	<0.00001	0%	0.36
	Nivolumab	3	0.86	0.63-1.18	0.35	78%	0.01
	Pembrolizumab	5	0.68	0.57-0.81	<0.0001	60%	0.04
PS 0	Total	9	0.74	0.64-0.84	<0.00001	35%	0.14
	1st Line	5	0.72	0.53-0.98	0.03	58%	0.05
	Nivolumab	1	1.11	0.74-1.66	0.62		
	Pembrolizumab	4	0.67	0.54-0.83	0.0002	38%	0.18
	≥2nd Line	4	0.72	0.59-0.88	0.001	0%	0.44
	Nivolumab	3	0.66	0.49-0.89	0.007	2%	0.36
	Pembrolizumab	1	0.78	0.60-1.01	0.06		
	monotherapy	7	0.79	0.68-0.91	0.001	3%	0.41
	Nivolumab	4	0.78	0.54-1.14	0.20	51%	0.10
	Pembrolizumab	3	0.78	0.65-0.94	0.008	0%	1.00
	combination therapy	2	0.48	0.33-0.69	0.0001	0%	0.63
	Pembrolizumab	2	0.48	0.33-0.69	0.0001	0%	0.63
	Nivolumab	4	0.78	0.54-1.14	0.20	51%	0.10
	Pembrolizumab	5	0.71	0.60-0.84	<0.0001	29%	0.23
PS 1	Total	7	0.65	0.57-0.75	<0.00001	51%	0.06
	1st Line	4	0.66	0.52-0.83	0.0005	66%	0.03
	Pembrolizumab	4	0.66	0.52-0.83	0.0005	66%	0.03
	≥2nd Line	3	0.63	0.56-0.72	<0.00001	0%	0.49
	Nivolumab	2	0.62	0.51-0.76	<0.00001	28%	0.24
	Pembrolizumab	1	0.64	0.54-0.76	<0.00001		
	monotherapy	5	0.67	0.57-0.79	<0.00001	58%	0.05
	Nivolumab	2	0.62	0.51-0.76	<0.00001	28%	0.24
	Pembrolizumab	3	0.69	0.55-0.87	0.001	70%	0.03
	combination therapy	2	0.59	0.47-0.74	<0.00001	0%	0.36
	Pembrolizumab	2	0.59	0.47-0.74	<0.00001	0%	0.36
	Nivolumab	2	0.62	0.51-0.76	<0.00001	28%	0.24
	Pembrolizumab	5	0.66	0.55-0.78	<0.00001	60%	0.04
Active or previous smoker	Total	6	0.67	0.60-0.74	<0.00001	7%	0.37
	1st Line	3	0.65	0.52-0.82	0.0003	51%	0.13
	Pembrolizumab	3	0.65	0.52-0.82	0.0003	51%	0.13
	≥2nd Line	3	0.68	0.59-0.79	<0.00001	0%	0.57
	Nivolumab	3	0.68	0.59-0.79	<0.00001	0%	0.57
	monotherapy	5	0.69	0.62-0.78	<0.00001	0%	0.62
	Nivolumab	3	0.68	0.59-0.79	<0.00001	0%	0.57
	Pembrolizumab	2	0.72	0.59-0.88	0.002	24%	0.25
	combination therapy	1	0.54	0.41-0.71	<0.0001		
	Pembrolizumab	1	0.54	0.41-0.71	<0.0001		

	Nivolumab	3	0.68	0.59-0.79	<0.00001	0%	0.57
	Pembrolizumab	3	0.65	0.52-0.82	0.0003	51%	0.13
Never smoker	Total	6	0.78	0.54-1.13	0.19	57%	0.04
	1st Line	4	0.69	0.35-1.37	0.29	71%	0.02
	Nivolumab	1	1.02	0.54-1.93	0.95		
	Pembrolizumab	3	0.57	0.18-1.80	0.34	80%	0.007
	≥2nd Line	2	0.85	0.60-1.21	0.38	25%	0.25
	Nivolumab	2	0.85	0.60-1.21	0.38	25%	0.25
	monotherapy	5	0.94	0.76-1.17	0.59	0%	0.77
	Nivolumab	3	0.89	0.66-1.21	0.46	0%	0.46
	Pembrolizumab	2	1.00	0.73-1.36	0.99	0%	0.92
	combination therapy	1	0.23	0.10-0.54	0.0007		
	Pembrolizumab	1	0.23	0.10-0.54	0.0007		
	Nivolumab	3	0.89	0.66-1.21	0.46	0%	0.46
	Pembrolizumab	3	0.57	0.18-1.80	0.34	80%	0.007
With brain metastases	Total	4	0.70	0.42-1.16	0.16	56%	0.08
	1st Line	2	0.44	0.27-0.70	0.0006	0%	0.40
	Pembrolizumab	2	0.44	0.27-0.70	0.0006	0%	0.40
	≥2nd Line	2	0.96	0.63-1.46	0.85	0%	0.59
	Nivolumab	2	0.96	0.63-1.46	0.85	0%	0.59
	monotherapy	3	0.93	0.63-1.39	0.74	0%	0.80
	Nivolumab	2	0.96	0.63-1.46	0.85	0%	0.59
	Pembrolizumab	1	0.73	0.20-2.62	0.63		
	combination therapy	1	0.41	0.24-0.67	0.0005		
	Pembrolizumab	1	0.41	0.24-0.67	0.0005		
	Nivolumab	2	0.96	0.63-1.46	0.85	0%	0.59
	Pembrolizumab	2	0.44	0.27-0.70	0.0006	0%	0.40
Without brain metastases	Total	5	0.65	0.58-0.73	<0.00001	0%	0.72
	1st Line	2	0.60	0.50-0.73	<0.00001	0%	0.70
	Pembrolizumab	2	0.60	0.50-0.73	<0.00001	0%	0.70
	≥2nd Line	3	0.68	0.59-0.78	<0.00001	0%	0.61
	Nivolumab	3	0.68	0.59-0.78	<0.00001	0%	0.61
	monotherapy	4	0.67	0.59-0.77	<0.00001	0%	0.77
	Nivolumab	3	0.68	0.59-0.78	<0.00001	0%	0.61
	Pembrolizumab	1	0.64	0.46-0.88	0.006		
	combination therapy	1	0.59	0.46-0.75	<0.0001		
	Pembrolizumab	1	0.59	0.46-0.75	<0.0001		
	Nivolumab	3	0.68	0.59-0.78	<0.00001	0%	0.61
	Pembrolizumab	2	0.60	0.50-0.73	<0.00001	0%	0.70
Liver metastases	Total	2	0.66	0.51-0.85	0.001	0%	0.74
	1st Line	1	0.62	0.39-0.98	0.04		
	Pembrolizumab	1	0.62	0.39-0.98	0.04		
	≥2nd Line	1	0.68	0.50-0.91	0.010		
	Nivolumab	1	0.68	0.50-0.91	0.010		
	monotherapy	1	0.68	0.50-0.91	0.010		
	Nivolumab	1	0.68	0.50-0.91	0.010		
	combination therapy	1	0.62	0.39-0.98	0.04		
	Pembrolizumab	1	0.62	0.39-0.98	0.04		
	Nivolumab	1	0.68	0.50-0.91	0.010		

	Pembrolizumab	1	0.62	0.39-0.98	0.04		
EGFR mutant	Total	2	1.04	0.70-1.53	0.85	0%	0.49
	1st Line	0					
	≥2nd Line	2	1.04	0.70-1.53	0.85	0%	0.49
	Nivolumab	1	1.18	0.69-2.00	0.55		
	Pembrolizumab	1	0.90	0.51-1.58	0.71		
	monotherapy	2	1.04	0.70-1.53	0.85	0%	0.49
	Nivolumab	1	1.18	0.69-2.00	0.55		
	Pembrolizumab	1	0.90	0.51-0.58	0.71		
	combination therapy	0					
	Nivolumab	1	1.18	0.69-2.00	0.55		
	Pembrolizumab	1	0.90	0.51-1.58	0.71		
EGFR wildtype	Total	2	0.68	0.60-0.78	<0.00001	0%	0.78
	1st Line	0					
	≥2nd Line	2	0.68	0.60-0.78	<0.00001	0%	0.78
	Nivolumab	1	0.66	0.51-0.86	0.002		
	Pembrolizumab	1	0.69	0.59-0.81	<0.00001		
	monotherapy	2	0.68	0.60-0.78	<0.00001	0%	0.78
	Nivolumab	1	0.66	0.51-0.86	0.002		
	Pembrolizumab	1	0.69	0.59-0.81	<0.00001		
	combination therapy	0					
	Nivolumab	1	0.66	0.51-0.86	0.002		
	Pembrolizumab	1	0.69	0.59-0.81	<0.00001		
East Asia	Total	4	0.69	0.57-0.83	0.0001	24%	0.27
	1st Line	3	0.69	0.54-0.90	0.005	49%	0.14
	Pembrolizumab	3	0.69	0.54-0.90	0.005	49%	0.14
	≥2nd Line	1	0.68	0.52-0.90	0.007		
	Nivolumab	1	0.68	0.52-0.90	0.007		
	monotherapy	3	0.71	0.59-0.87	0.0007	12%	0.32
	Nivolumab	1	0.68	0.52-0.90	0.007		
	Pembrolizumab	2	0.62	0.30-1.28	0.20	52%	0.15
	combination therapy	1	0.44	0.22-0.89	0.02		
	Pembrolizumab	1	0.44	0.22-0.89	0.02		
	Nivolumab	1	0.68	0.52-0.90	0.007		
	Pembrolizumab	3	0.69	0.54-0.90	0.005	49%	0.14
Europe	Total	2	0.64	0.40-1.03	0.07	76%	0.04
	1st Line	0					
	≥2nd Line	2	0.64	0.40-1.03	0.07	76%	0.04
	Nivolumab	2	0.64	0.40-1.03	0.07	76%	0.04
	monotherapy	2	0.64	0.40-1.03	0.07	76%	0.04
	Nivolumab	2	0.64	0.40-1.03	0.07		
	combination therapy	0					
	Nivolumab	2	0.64	0.40-1.03	0.07	76%	0.04
	Pembrolizumab	0					
US/Canada	Total	2	0.54	0.41-0.71	<0.0001	0%	0.65
	1st Line	0					
	≥2nd Line	2	0.54	0.41-0.71	<0.0001	0%	0.65
	Nivolumab	2	0.54	0.41-0.71	<0.0001	0%	0.65
	monotherapy	2	0.54	0.41-0.71	<0.0001	0%	0.65

	Nivolumab	2	0.54	0.41-0.71	<0.0001		
	combination therapy	0					
	Nivolumab	2	0.54	0.41-0.71	<0.0001	0%	0.65
	Pembrolizumab	0					
PD-L1 tumor proportion score <1%	Total	5	0.69	0.58-0.81	<0.0001	37%	0.18
	1st Line	2	0.55	0.41-0.73	<0.0001	0%	0.58
	Pembrolizumab	2	0.55	0.41-0.73	<0.0001	0%	0.58
	≥2nd Line	3	0.77	0.63-0.96	0.02	18%	0.30
	Nivolumab	3	0.77	0.63-0.96	0.02	18%	0.30
	monotherapy	3	0.77	0.63-0.96	0.02	18%	0.30
	Nivolumab	3	0.77	0.63-0.96	0.02	18%	0.30
	combination therapy	2	0.55	0.41-0.73	<0.0001	0%	0.58
	Pembrolizumab	2	0.55	0.41-0.73	<0.0001	0%	0.58
	Nivolumab	3	0.77	0.63-0.96	0.02	18%	0.30
	Pembrolizumab	2	0.55	0.41-0.73	<0.0001	0%	0.58
PD-L1 tumor proportion score ≥1%	Total	7	0.71	0.66-0.77	<0.00001	17%	0.30
	1st Line	3	0.71	0.58-0.87	0.001	53%	0.12
	Pembrolizumab	3	0.71	0.58-0.87	0.001	53%	0.12
	≥2nd Line	4	0.67	0.60-0.75	<0.00001	0%	0.82
	Nivolumab	3	0.63	0.51-0.77	<0.00001	0%	0.86
	Pembrolizumab	1	0.69	0.60-0.80	<0.00001		
	monotherapy	5	0.73	0.67-0.80	<0.00001	26%	0.25
	Nivolumab	3	0.63	0.51-0.77	<0.00001	0%	0.86
	Pembrolizumab	2	0.75	0.64-0.88	0.0003	60%	0.11
	combination therapy	2	0.62	0.50-0.77	<0.0001	0%	0.78
	Pembrolizumab	2	0.62	0.50-0.77	<0.00001	0%	0.78
	Nivolumab	3	0.63	0.51-0.77	<0.00001	0%	0.86
	Pembrolizumab	4	0.73	0.67-0.80	<0.00001	41%	0.17
PD-L1 tumor proportion score 1-49%	Total	4	0.77	0.63-0.93	0.007	51%	0.10
	1st Line	3	0.72	0.52-1.01	0.06	67%	0.05
	Pembrolizumab	3	0.72	0.52-1.01	0.06	67%	0.05
	≥2nd Line	1	0.78	0.65-0.94	0.009		
	Pembrolizumab	1	0.78	0.65-0.94	0.009		
	monotherapy	2	0.85	0.75-0.97	0.01	38%	0.21
	Pembrolizumab	2	0.85	0.75-0.97	0.01	38%	0.21
	combination therapy	2	0.60	0.44-0.81	0.0007	0%	0.77
	Pembrolizumab	2	0.60	0.44-0.81	0.0007	0%	0.77
	Nivolumab	0					
	Pembrolizumab	4	0.77	0.63-0.93	0.007	51%	0.10
PD-L1 tumor proportion score ≥50%	Total	6	0.64	0.57-0.72	<0.00001	29%	0.22
	1st Line	5	0.69	0.60-0.79	<0.00001	0%	0.54
	Nivolumab	1	0.90	0.63-1.29	0.57		
	Pembrolizumab	4	0.66	0.56-0.76	<0.00001	0%	0.90
	≥2nd Line	1	0.53	0.42-0.66	<0.00001		
	Pembrolizumab	1	0.53	0.42-0.66	<0.00001		
	monotherapy	4	0.66	0.54-0.80	<0.0001	56%	0.08
	Nivolumab	1	0.90	0.63-1.29	0.57		
	Pembrolizumab	3	0.61	0.54-0.70	<0.00001	34%	0.22
	combination therapy	2	0.60	0.44-0.84	0.002	0%	0.81

Pembrolizumab	2	0.60	0.44-0.84	0.002	0%	0.81
Nivolumab	1	0.90	0.63-1.29	0.57		
Pembrolizumab	5	0.61	0.54-0.70	<0.00001	0%	0.54

Table S2 Analyses of PFS in subgroups of patients with varying clinical and molecular features

Population	Subgroup	No. of studies	Test of association			Test of heterogeneity	
			HR	CI95%	P value	I^2	P value
Aged <65 years	Total	8	0.71	0.56-0.89	0.003	81%	<0.00001
	1st Line	4	0.63	0.39-1.01	0.06	89%	<0.00001
	Nivolumab	1	1.17	0.88-1.56	0.28		
	Pembrolizumab	3	0.48	0.40-0.58	<0.00001	1%	0.36
	≥2nd Line	4	0.81	0.72-0.92	0.0008	0%	0.42
	Nivolumab	3	0.80	0.68-0.93	0.004	24%	0.27
	Pembrolizumab	1	0.84	0.69-1.02	0.08		
	monotherapy	6	0.83	0.70-0.97	0.02	53%	0.06
	Nivolumab	4	0.86	0.68-1.08	0.19	63%	0.04
	Pembrolizumab	2	0.79	0.66-0.94	0.009	48%	0.17
	combination therapy	2	0.46	0.37-0.56	<0.00001	0%	0.43
	Pembrolizumab	2	0.46	0.37-0.56	<0.00001	0%	0.43
	Nivolumab	4	0.86	0.68-1.08	0.19	63%	0.04
	Pembrolizumab	4	0.58	0.41-0.82	0.002	83%	0.0004
Aged ≥65 years	Total	6	0.76	0.58-0.99	0.04	74%	0.002
	1st Line	4	0.73	0.49-1.07	0.11	83%	0.0006
	Nivolumab	1	1.21	0.91-1.62	0.19		
	Pembrolizumab	3	0.63	0.52-0.76	<0.00001	42%	0.18
	≥2nd Line	2	0.87	0.69-1.08	0.21	19%	0.27
	Nivolumab	1	0.68	0.42-1.10	0.12		
	Pembrolizumab	1	0.93	0.72-1.19	0.55		
	monotherapy	4	0.79	0.54-1.17	0.24	80%	0.002
	Nivolumab	2	0.94	0.53-1.65	0.83	76%	0.04
	Pembrolizumab	2	0.66	0.33-1.34	0.25	87%	0.005
	combination therapy	2	0.68	0.55-0.84	0.0004	0%	0.42
	Pembrolizumab	2	0.68	0.55-0.84	0.0004	0%	0.42
	Nivolumab	2	0.94	0.53-1.65	0.83	76%	0.04
	Pembrolizumab	4	0.69	0.52-0.91	0.008	67%	0.03
Aged 65-74 years	Total	2	0.71	0.40-1.28	0.26	77%	0.04
	1st Line	0					
	≥2nd Line	2	0.71	0.40-1.28	0.26	77%	0.04
	Nivolumab	2	0.71	0.40-1.28	0.26	77%	0.04
	monotherapy	2	0.71	0.40-1.28	0.26	77%	0.04
	Nivolumab	2	0.71	0.40-1.28	0.26	77%	0.04
	combination therapy	0					
	Nivolumab	2	0.71	0.40-1.28	0.26	77%	0.04
	Pembrolizumab	0					
Aged ≥75 years	Total	2	1.24	0.73-2.11	0.43	13%	0.28
	1st Line	0					
	≥2nd Line	2	1.24	0.73-2.11	0.43	13%	0.28
	Nivolumab	2	1.24	0.73-2.11	0.43	13%	0.28
	monotherapy	2	1.24	0.73-2.11	0.43	13%	0.28
	Nivolumab	2	1.24	0.73-2.11	0.43	13%	0.28
	combination therapy	0					

	Nivolumab	2	1.24	0.73-2.11	0.43	13%	0.28
	Pembrolizumab	0					
Male	Total	8	0.69	0.58-0.82	<0.0001	70%	0.002
	1st Line	4	0.64	0.44-0.92	0.02	85%	0.0002
	Nivolumab	1	1.05	0.81-1.37	0.70		
	Pembrolizumab	3	0.55	0.43-0.71	<0.00001	57%	0.10
	≥2nd Line	4	0.74	0.66-0.84	<0.00001	0%	0.57
	Nivolumab	3	0.72	0.62-0.84	<0.0001	0%	0.43
	Pembrolizumab	1	0.78	0.64-0.94	0.010		
	monotherapy	6	0.72	0.58-0.89	0.002	73%	0.002
	Nivolumab	4	0.79	0.64-0.97	0.03	61%	0.05
	Pembrolizumab	2	0.56	0.29-1.10	0.09	89%	0.002
	combination therapy	2	0.61	0.51-0.73	<0.00001	0%	0.48
	Pembrolizumab	2	0.61	0.51-0.73	<0.00001	0%	0.48
	Nivolumab	4	0.79	0.64-0.97	0.03	61%	0.05
	Pembrolizumab	4	0.61	0.47-0.77	<0.0001	72%	0.01
Female	Total	8	0.80	0.59-1.10	0.17	83%	<0.00001
	1st Line	4	0.67	0.36-1.25	0.21	90%	<0.00001
	Nivolumab	1	1.36	0.98-1.90	0.07		
	Pembrolizumab	3	0.51	0.35-0.74	0.0004	57%	0.10
	≥2nd Line	4	1.00	0.84-1.18	0.98	0%	0.68
	Nivolumab	3	0.99	0.80-1.22	0.90	0%	0.48
	Pembrolizumab	1	1.02	0.78-1.32	0.91		
	monotherapy	6	1.03	0.89-1.19	0.68	18%	0.30
	Nivolumab	4	1.08	0.91-1.30	0.37	26%	0.25
	Pembrolizumab	2	0.95	0.75-1.19	0.64	17%	0.27
	combination therapy	2	0.42	0.32-0.55	<0.00001	0%	0.46
	Pembrolizumab	2	0.42	0.32-0.55	<0.00001	0%	0.46
	Nivolumab	4	1.08	0.91-1.30	0.37	26%	0.25
	Pembrolizumab	4	0.62	0.38-1.03	0.07	86%	<0.0001
Squamous	Total	6	0.63	0.56-0.72	<0.00001	44%	0.11
	1st Line	3	0.58	0.40-0.85	0.005	58%	0.09
	Nivolumab	1	0.83	0.54-1.26	0.38		
	Pembrolizumab	2	0.54	0.44-0.66	<0.00001	37%	0.21
	≥2nd Line	3	0.68	0.57-0.82	<0.0001	30%	0.24
	Nivolumab	2	0.61	0.49-0.76	<0.0001	0%	0.93
	Pembrolizumab	1	0.86	0.62-1.20	0.38		
	monotherapy	5	0.68	0.57-0.80	<0.00001	44%	0.13
	Nivolumab	3	0.65	0.54-0.79	<0.0001	0%	0.46
	Pembrolizumab	2	0.58	0.24-1.40	0.23	80%	0.02
	combination therapy	1	0.56	0.45-0.70	<0.00001		
	Pembrolizumab	1	0.56	0.45-0.70	<0.00001		
	Nivolumab	3	0.65	0.54-0.79	<0.0001	0%	0.46
	Pembrolizumab	3	0.60	0.40-0.90	0.01	72%	0.03
Non-squamous	Total	7	0.75	0.58-0.99	0.04	89%	<0.00001
	1st Line	4	0.66	0.39-1.12	0.12	93%	<0.00001

	Nivolumab	1	1.29	1.02-1.63	0.03		
	Pembrolizumab	3	0.50	0.43-0.58	<0.00001	0%	0.77
	≥2nd Line	3	0.89	0.79-0.99	0.04	0%	0.84
	Nivolumab	2	0.91	0.78-1.05	0.19	0%	0.72
	Pembrolizumab	1	0.86	0.71-1.03	0.10		
	monotherapy	5	0.88	0.71-1.10	0.26	78%	0.001
	Nivolumab	3	1.01	0.81-1.27	0.93	69%	0.04
	Pembrolizumab	2	0.70	0.45-1.08	0.11	81%	0.02
	combination therapy	2	0.49	0.41-0.58	<0.00001	0%	0.64
	Pembrolizumab	2	0.49	0.41-0.58	<0.00001	0%	0.64
	Nivolumab	3	1.01	0.81-1.27	0.93	69%	0.04
	Pembrolizumab	4	0.60	0.43-0.83	0.002	85%	0.0002
PS 0	Total	8	0.76	0.53-1.10	0.15	85%	<0.00001
	1st Line	4	0.64	0.32-1.28	0.21	91%	<0.00001
	Nivolumab	1	1.69	1.18-2.42	0.004		
	Pembrolizumab	3	0.47	0.37-0.59	<0.00001	0%	0.93
	≥2nd Line	4	0.93	0.69-1.25	0.63	53%	0.09
	Nivolumab	3	0.85	0.54-1.33	0.48	62%	0.07
	Pembrolizumab	1	1.08	0.82-1.43	0.57		
	monotherapy	6	0.91	0.64-1.31	0.63	78%	0.0003
	Nivolumab	4	1.02	0.64-1.63	0.94	78%	0.004
	Pembrolizumab	2	0.72	0.30-1.70	0.45	88%	0.005
	combination therapy	2	0.47	0.36-0.61	<0.00001	0%	0.74
	Pembrolizumab	2	0.47	0.36-0.61	<0.00001	0%	0.74
	Nivolumab	4	1.02	0.64-1.63	0.94	78%	0.004
	Pembrolizumab	4	0.58	0.35-0.95	0.03	85%	0.0001
PS 1	Total	6	0.65	0.59-0.72	<0.00001	24%	0.25
	1st Line	3	0.57	0.49-0.67	<0.00001	0%	0.68
	Pembrolizumab	3	0.57	0.49-0.67	<0.00001	0%	0.68
	≥2nd Line	3	0.71	0.62-0.81	<0.00001	0%	0.50
	Nivolumab	2	0.66	0.55-0.81	<0.0001	0%	0.49
	Pembrolizumab	1	0.76	0.63-0.91	0.003		
	monotherapy	4	0.68	0.60-0.78	<0.00001	31%	0.23
	Nivolumab	2	0.66	0.55-0.81	<0.0001	0%	0.49
	Pembrolizumab	2	0.64	0.43-0.94	0.02	73%	0.05
	combination therapy	2	0.59	0.49-0.70	<0.00001	0%	0.60
	Pembrolizumab	2	0.59	0.49-0.70	<0.00001	0%	0.60
	Nivolumab	2	0.66	0.55-0.81	<0.0001	0%	0.49
	Pembrolizumab	4	0.64	0.57-0.73	<0.00001	50%	0.11
Active or previous smoker	Total	4	0.67	0.55-0.82	0.0001	66%	0.03
	1st Line	1	0.54	0.43-0.66	<0.00001		
	Pembrolizumab	1	0.54	0.43-0.66	<0.00001		
	≥2nd Line	3	0.74	0.65-0.86	<0.0001	19%	0.29
	Nivolumab	3	0.74	0.65-0.86	<0.0001	19%	0.29

	monotherapy	3	0.74	0.65-0.86	<0.0001	19%	0.29
	Nivolumab	3	0.74	0.65-0.86	<0.0001	19%	0.29
	combination therapy	1	0.54	0.43-0.66	<0.00001		
	Pembrolizumab	1	0.54	0.43-0.66	<0.00001		
	Nivolumab	3	0.74	0.65-0.86	<0.0001	19%	0.29
	Pembrolizumab	1	0.54	0.43-0.66	<0.00001		
Never smoker	Total	5	1.06	0.60-1.86	0.85	76%	0.002
	1st Line	3	1.01	0.25-4.06	0.99	86%	0.0007
	Nivolumab	1	2.51	1.31-4.83	0.006		
	Pembrolizumab	2	0.46	0.25-0.84	0.01	0%	0.52
	≥2nd Line	2	1.09	0.69-1.72	0.71	59%	0.12
	Nivolumab	2	1.09	0.69-1.72	0.71	59%	0.12
	monotherapy	4	1.34	0.82-2.20	0.25	62%	0.05
	Nivolumab	3	1.38	0.80-2.38	0.25	74%	0.02
	Pembrolizumab	1	0.90	0.11-7.59	0.92		
	combination therapy	1	0.43	0.23-0.81	0.009		
	Pembrolizumab	1	0.43	0.23-0.81	0.009		
	Nivolumab	3	1.38	0.80-2.38	0.25	74%	0.02
	Pembrolizumab	2	0.46	0.25-0.84	0.01	0%	0.52
With brain metastases	Total	4	0.57	0.43-0.76	0.0001	9%	0.35
	1st Line	2	0.44	0.29-0.67	0.0001	0%	0.64
	Pembrolizumab	2	0.44	0.29-0.67	0.0001	0%	0.64
	≥2nd Line	2	0.71	0.48-1.04	0.08	0%	0.51
	Nivolumab	2	0.71	0.48-1.04	0.08	0%	0.51
	monotherapy	3	0.69	0.48-0.99	0.04	0%	0.73
	Nivolumab	2	0.71	0.48-1.04	0.08	0%	0.51
	Pembrolizumab	1	0.55	0.20-1.56	0.26		
	combination therapy	1	0.42	0.27-0.67	0.0002		
	Pembrolizumab	1	0.42	0.27-0.67	0.0002		
	Nivolumab	2	0.71	0.48-1.04	0.08	0%	0.51
	Pembrolizumab	2	0.44	0.29-0.67	0.0001	0%	0.64
Without brain metastases	Total	5	0.65	0.50-0.85	0.002	85%	<0.0001
	1st Line	2	0.49	0.41-0.58	<0.00001	0%	0.85
	Pembrolizumab	2	0.49	0.41-0.58	<0.00001	0%	0.85
	≥2nd Line	3	0.79	0.65-0.97	0.02	55%	0.11
	Nivolumab	3	0.79	0.65-0.97	0.02	55%	0.11
	monotherapy	4	0.71	0.55-0.91	0.008	76%	0.006
	Nivolumab	3	0.79	0.65-0.97	0.02	55%	0.11
	Pembrolizumab	1	0.50	0.36-0.68	<0.0001		
	combination therapy	1	0.48	0.39-0.59	<0.00001		
	Pembrolizumab	1	0.48	0.39-0.59	<0.00001		
	Nivolumab	3	0.79	0.65-0.97	0.02	55%	011
	Pembrolizumab	2	0.49	0.41-0.58	<0.00001	0%	0.85
EGFR mutant	Total	3	1.10	0.50-2.42	0.81	71%	0.03
	1st Line	0					
	≥2nd Line	3	1.10	0.50-2.42	0.81	71%	0.03

	Nivolumab	1	1.46	0.90-2.37	0.13		
	Pembrolizumab	2	0.75	0.11-4.90	0.76	85%	0.010
	monotherapy	2	1.57	1.07-2.32	0.02	0%	0.62
	Nivolumab	1	1.46	0.90-2.37	0.13		
	Pembrolizumab	1	1.79	0.94-3.42	0.08		
	combination therapy	1	0.26	0.07-0.97	0.04		
	Pembrolizumab	1	0.26	0.07-0.97	0.04		
	Nivolumab	1	1.46	0.90-2.37	0.13		
	Pembrolizumab	2	0.75	0.11-4.90	0.76	85%	0.010
EGFR	Total	3	0.69	0.48-0.99	0.05	78%	0.01
wildtype	1st Line	0					
	≥2nd Line	3	0.69	0.48-0.99	0.05	78%	0.01
	Nivolumab	1	0.83	0.65-1.06	0.14		
	Pembrolizumab	2	0.50	0.16-1.53	0.22	89%	0.003
	monotherapy	2	0.83	0.73-0.95	0.008	0%	0.97
	Nivolumab	1	0.83	0.65-1.06	0.14		
	Pembrolizumab	1	0.83	0.71-0.98	0.03		
	combination therapy	1	0.26	0.13-0.55	0.0004		
	Pembrolizumab	1	0.26	0.13-0.55	0.0004		
	Nivolumab	1	0.83	0.65-1.06	0.14		
	Pembrolizumab	2	0.50	0.16-1.53	0.22	89%	0.003
East Asia	Total	2	0.46	0.29-0.71	0.0006	0%	0.54
	1st Line	2	0.46	0.29-0.71	0.0006	0%	0.54
	Pembrolizumab	2	0.46	0.29-0.71	0.0006	0%	0.54
	≥2nd Line	0					
	monotherapy	1	0.35	0.14-0.91	0.03		
	Pembrolizumab	1	0.35	0.14-0.91	0.03		
	combination therapy	1	0.49	0.30-0.82	0.006		
	Pembrolizumab	1	0.49	0.30-0.82	0.006		
	Nivolumab	0					
	Pembrolizumab	2	0.46	0.29-0.71	0.0006	0%	0.54
Europe	Total	2	0.78	0.43-1.39	0.39	86%	0.008
	1st Line	0					
	≥2nd Line	2	0.78	0.43-1.39	0.39	86%	0.008
	Nivolumab	2	0.78	0.43-1.39	0.39	86%	0.008
	monotherapy	2	0.78	0.43-1.39	0.39	86%	0.008
	Nivolumab	2	0.78	0.43-1.39	0.39	86%	0.008
	combination therapy	0					
	Nivolumab	2	0.78	0.43-1.39	0.39	86%	0.008
	Pembrolizumab	0					
US/Canada	Total	2	0.65	0.50-0.84	0.001	0%	0.85
	1st Line	0					
	≥2nd Line	2	0.65	0.50-0.84	0.001	0%	0.85
	Nivolumab	2	0.65	0.50-0.84	0.001	0%	0.85
	monotherapy	2	0.65	0.50-0.84	0.001	0%	0.85
	Nivolumab	2	0.65	0.50-0.84	0.001	0%	0.85

	combination therapy	0					
	Nivolumab	2	0.65	0.50-0.84	0.001	0%	0.85
	Pembrolizumab	0					
PD-L1 tumor proportion score <1%	Total	6	0.74	0.58-0.95	0.02	58%	0.04
	1st Line	2	0.66	0.52-0.84	0.0008	0%	0.84
	Pembrolizumab	2	0.66	0.52-0.84	0.0008	0%	0.84
	≥2nd Line	4	0.78	0.54-1.14	0.20	67%	0.03
	Nivolumab	3	0.85	0.59-1.23	0.39	70%	0.04
	Pembrolizumab	1	0.41	0.16-1.05	0.06		
	monotherapy	3	0.85	0.59-1.23	0.39	70%	0.04
	Nivolumab	3	0.85	0.59-1.23	0.39	70%	0.04
	combination therapy	3	0.64	0.51-0.81	0.0002	0%	0.62
	Pembrolizumab	3	0.64	0.51-0.81	0.0002	0%	0.62
	Nivolumab	3	0.85	0.59-1.23	0.39	70%	0.04
	Pembrolizumab	3	0.64	0.51-0.81	0.0002	0%	0.62
PD-L1 tumor proportion score ≥1%	Total	7	0.60	0.42-0.86	0.005	91%	<0.00001
	1st Line	3	0.61	0.31-1.18	0.14	97%	<0.00001
	Pembrolizumab	3	0.61	0.31-1.18	0.14	97%	<0.00001
	≥2nd Line	4	0.66	0.49-0.88	0.004	53%	0.10
	Nivolumab	3	0.72	0.60-0.86	0.0003	0%	0.88
	Pembrolizumab	1	0.16	0.05-0.52	0.002		
	monotherapy	4	0.81	0.62-1.05	0.12	77%	0.005
	Nivolumab	3	0.72	0.60-0.86	0.0003	0%	0.88
	Pembrolizumab	1	1.07	0.94-1.21	0.32		
	combination therapy	3	0.44	0.37-0.52	<0.00001	47%	0.15
	Pembrolizumab	3	0.44	0.37-0.52	<0.00001	47%	0.15
	Nivolumab	3	0.72	0.60-0.86	0.0003	0%	0.88
	Pembrolizumab	4	0.50	0.26-0.94	0.03	96%	<0.00001
PD-L1 tumor proportion score 1-49%	Total	3	0.68	0.41-1.12	0.13	88%	0.0003
	1st Line	2	0.53	0.42-0.69	<0.00001	0%	0.74
	Pembrolizumab	2	0.53	0.42-0.69	<0.00001	0%	0.74
	≥2nd Line	1	1.04	0.85-1.27	0.71		
	Pembrolizumab	1	1.04	0.85-1.27	0.71		
	monotherapy	1	1.04	0.85-1.27	0.71		
	Pembrolizumab	1	1.04	0.85-1.27	0.71		
	combination therapy	2	0.53	0.42-0.69	<0.00001	0%	0.74
	Pembrolizumab	2	0.53	0.42-0.69	<0.00001	0%	0.74
	Nivolumab	0					
	Pembrolizumab	3	0.68	0.41-1.12	0.13	88%	0.0003
PD-L1 tumor proportion score ≥50%	Total	6	0.58	0.43-0.79	0.0006	86%	<0.00001
	1st Line	5	0.58	0.39-0.86	0.007	88%	<0.00001
	Nivolumab	1	1.07	0.77-1.49	0.68		
	Pembrolizumab	4	0.50	0.32-0.76	0.001	87%	<0.0001
	≥2nd Line	1	0.59	0.46-0.74	<0.00001		
	Pembrolizumab	1	0.59	0.46-0.74	<0.00001		
	monotherapy	4	0.71	0.53-0.95	0.02	81%	0.001

Nivolumab	1	1.07	0.77-1.49	0.68		
Pembrolizumab	3	0.63	0.47-0.84	0.002	77%	0.01
combination therapy	2	0.37	0.28-0.48	<0.00001	0%	0.93
Pembrolizumab	2	0.37	0.28-0.48	<0.00001	0%	0.93
Nivolumab	1	1.07	0.77-1.49	0.68		
Pembrolizumab	5	0.52	0.38-0.71	<0.0001	83%	<0.0001

Table S3 Deep analyses of OS in subgroups of patients with varying clinical and molecular features

Population	Line	Regimen	No. of studies	Test of association			Test of heterogeneity	
				HR	CI95%	P value	I^2	P value
Aged <65 years	1st Line	P combined therapy	2	0.47	0.36-0.61	<0.00001	0%	0.51
		P monotherapy	2	0.78	0.65-0.93	0.005	24%	0.25
		N combined therapy	0					
		N monotherapy	1	1.13	0.83-1.54	0.44		
	≥2nd Line	P combined therapy	0					
		P monotherapy	1	0.62	0.51-0.75	<0.00001		
		N combined therapy	0					
		N monotherapy	3	0.72	0.62-0.85	0.0001	49%	0.14
Aged ≥65 years	1st Line	P combined therapy	2	0.69	0.53-0.91	0.007	0%	0.60
		P monotherapy	1	0.64	0.42-0.98	0.04		
		N combined therapy	0					
		N monotherapy	1	1.04	0.77-1.41	0.79		
	≥2nd Line	P combined therapy	0					
		P monotherapy	1	0.79	0.63-1.00	0.05		
		N combined therapy	0					
		N monotherapy	1	0.50	0.29-0.85	0.01		
Aged 65-74 years	1st Line	P combined therapy	0					
		P monotherapy	0					
		N combined therapy	0					
		N monotherapy	0					
	≥2nd Line	P combined therapy	0					
		P monotherapy	0					
		N combined therapy	0					
		N monotherapy	2	0.61	0.46-0.80	0.0005	0%	0.67
Aged ≥75 years	1st Line	P combined therapy	0					
		P monotherapy	2	0.82	0.56-1.21	0.32	10%	0.29
		N combined therapy	0					
		N monotherapy	0					
	≥2nd Line	P combined therapy	0					
		P monotherapy	1	0.72	0.43-1.21	0.21		
		N combined therapy	0					
		N monotherapy	2	1.20	0.68-2.12	0.52	34%	0.22
Male	1st Line	P combined therapy	2	0.70	0.56-0.88	0.002	0%	0.95
		P monotherapy	2	0.68	0.46-1.00	0.05	71%	0.06
		N combined therapy	0					
		N monotherapy	1	0.97	0.74-1.26	0.80		
	≥2nd Line	P combined therapy	0					
		P monotherapy	1	0.70	0.58-0.84	0.0001		
		N combined therapy	0					
		N monotherapy	3	0.68	0.57-0.80	<0.00001	0%	0.44
Female	1st Line	P combined therapy	2	0.32	0.23-0.46	<0.00001	0%	0.34
		P monotherapy	2	0.90	0.71-1.15	0.41	0%	0.83
		N combined therapy	0					
		N monotherapy	1	1.15	0.79-1.66	0.47		
	≥2nd Line	P combined therapy	0					
		P monotherapy	1	0.66	0.52-0.83	0.0004		
		N combined therapy	0					

		N monotherapy	3	0.75	0.59-0.96	0.02	0%	0.91
Squamous	1st Line	P combined therapy	1	0.64	0.49-0.85	0.002		
		P monotherapy	2	0.74	0.61-0.92	0.005	0%	0.94
		N combined therapy	0					
		N monotherapy	1	0.82	0.54-1.24	0.34		
	≥2nd Line	P combined therapy	0					
		P monotherapy	1	0.84	0.61-1.15	0.27		
		N combined therapy	0					
		N monotherapy	2	0.60	0.47-0.75	<0.0001	0%	0.88
Non-squamous	1st Line	P combined therapy	2	0.59	0.48-0.72	<0.00001	0%	0.36
		P monotherapy	2	0.73	0.50-1.07	0.10	73%	0.05
		N combined therapy	0					
		N monotherapy	1	1.17	0.91-1.52	0.23		
	≥2nd Line	P combined therapy	0					
		P monotherapy	1	0.68	0.57-0.81	<0.0001		
		N combined therapy	0					
		N monotherapy	2	0.73	0.62-0.87	0.0003	0%	0.78
PS 0	1st Line	P combined therapy	2	0.48	0.33-0.69	0.0001	0%	0.63
		P monotherapy	2	0.78	0.61-1.01	0.06	0%	0.97
		N combined therapy	0					
		N monotherapy	1	1.11	0.74-1.66	0.62		
	≥2nd Line	P combined therapy	0					
		P monotherapy	1	0.78	0.60-1.01	0.06		
		N combined therapy	0					
		N monotherapy	3	0.66	0.49-0.89	0.007	2%	0.36
PS 1	1st Line	P combined therapy	2	0.59	0.47-0.74	<0.00001	0%	0.36
		P monotherapy	2	0.71	0.48-1.04	0.08	73%	0.05
		N combined therapy	0					
		N monotherapy	0					
	≥2nd Line	P combined therapy	0					
		P monotherapy	1	0.64	0.54-0.76	<0.00001		
		N combined therapy	0					
		N monotherapy	2	0.62	0.51-0.76	<0.00001	28%	0.24
Active or previous smoker	1st Line	P combined therapy	1	0.54	0.41-0.71	<0.0001		
		P monotherapy	2	0.72	0.59-0.88	0.002	24%	0.25
		N combined therapy	0					
		N monotherapy	0					
	≥2nd Line	P combined therapy	0					
		P monotherapy	0					
		N combined therapy	0					
		N monotherapy	3	0.68	0.59-0.79	<0.00001	0%	0.57
Never smoker	1st Line	P combined therapy	1	0.23	0.10-0.54	0.0007		
		P monotherapy	2	1.00	0.73-1.36	0.99	0%	0.92
		N combined therapy	0					
		N monotherapy	1	1.02	0.54-1.93	0.95		
	≥2nd Line	P combined therapy	0					
		P monotherapy	0					
		N combined therapy	0					
		N monotherapy	2	0.85	0.60-1.21	0.38	25%	0.25

With brain metastases	1st Line	P combined therapy	1	0.41	0.24-0.67	0.0005		
		P monotherapy	1	0.73	0.20-2.62	0.63		
		N combined therapy	0					
		N monotherapy	0					
	≥2nd Line	P combined therapy	0					
		P monotherapy	0					
		N combined therapy	0					
		N monotherapy	2	0.96	0.63-1.46	0.85	0%	0.59
Without brain metastases	1st Line	P combined therapy	1	0.59	0.46-0.75	<0.0001		
		P monotherapy	1	0.64	0.46-0.88	0.006		
		N combined therapy	0					
		N monotherapy	0					
	≥2nd Line	P combined therapy	0					
		P monotherapy	0					
		N combined therapy	0					
		N monotherapy	3	0.68	0.59-0.78	<0.00001	0%	0.61
Liver metastases	1st Line	P combined therapy	1	0.62	0.39-0.98	0.04		
		P monotherapy	0					
		N combined therapy	0					
		N monotherapy	0					
	≥2nd Line	P combined therapy	0					
		P monotherapy	0					
		N combined therapy	0					
		N monotherapy	1	0.68	0.50-0.91	0.010		
EGFR mutan	1st Line	P combined therapy	0					
		P monotherapy	0					
		N combined therapy	0					
		N monotherapy	0					
	≥2nd Line	P combined therapy	0					
		P monotherapy	1	0.90	0.51-1.58	0.71		
		N combined therapy	0					
		N monotherapy	1	1.18	0.69-2.00	0.55		
EGFR wildtype	1st Line	P combined therapy	0					
		P monotherapy	0					
		N combined therapy	0					
		N monotherapy	0					
	≥2nd Line	P combined therapy	0					
		P monotherapy	1	0.69	0.59-0.81	<0.00001		
		N combined therapy	0					
		N monotherapy	1	0.66	0.51-0.86	0.002		
East Asia	1st Line	P combined therapy	1	0.44	0.22-0.89	0.02		
		P monotherapy	2	0.62	0.30-1.28	0.20	52%	0.15
		N combined therapy	0					
		N monotherapy	0					
	≥2nd Line	P combined therapy	0					
		P monotherapy	0					
		N combined therapy	0					
		N monotherapy	1	0.68	0.52-0.90	0.007		
Europe	1st Line	P combined therapy	0					

		P monotherapy	0					
		N combined therapy	0					
		N monotherapy	0					
	≥2nd Line	P combined therapy	0					
		P monotherapy	0					
		N combined therapy	0					
		N monotherapy	2	0.64	0.40-1.03	0.07	76%	0.04
US/Canada	1st Line	P combined therapy	0					
		P monotherapy	0					
		N combined therapy	0					
		N monotherapy	0					
	≥2nd Line	P combined therapy	0					
		P monotherapy	0					
		N combined therapy	0					
		N monotherapy	2	0.54	0.41-0.71	<0.0001	0%	0.65
PD-L1 tumor proportion score <1%	1st Line	P combined therapy	2	0.55	0.41-0.73	<0.0001	0%	0.58
		P monotherapy	0					
		N combined therapy	0					
		N monotherapy	0					
	≥2nd Line	P combined therapy	0					
		P monotherapy	0					
		N combined therapy	0					
		N monotherapy	3	0.77	0.63-0.96	0.02	18%	0.30
PD-L1 tumor proportion score ≥1%	1st Line	P combined therapy	2	0.62	0.50-0.77	<0.0001	0%	0.78
		P monotherapy	1	0.81	0.71-0.93	0.003		
		N combined therapy	0					
		N monotherapy	0					
	≥2nd Line	P combined therapy	0					
		P monotherapy	1	0.69	0.60-0.80	<0.00001		
		N combined therapy	0					
		N monotherapy	3	0.63	0.51-0.77	<0.00001	0%	0.86
PD-L1 tumor proportion score 1-49%	1st Line	P combined therapy	2	0.60	0.44-0.81	0.0007	0%	0.77
		P monotherapy	1	0.92	0.77-1.11	0.40		
		N combined therapy	0					
		N monotherapy	0					
	≥2nd Line	P combined therapy	0					
		P monotherapy	1	0.78	0.65-0.94	0.009		
		N combined therapy	0					
		N monotherapy	0					
PD-L1 tumor proportion score ≥50%	1st Line	P combined therapy	2	0.60	0.44-0.84	0.002	0%	0.81
		P monotherapy	2	0.67	0.57-0.80	<0.00001	0%	0.66
		N combined therapy	0					
		N monotherapy	1	0.90	0.63-1.29	0.57		
	≥2nd Line	P combined therapy	0					
		P monotherapy	1	0.53	0.42-0.66	<0.00001		
		N combined therapy	0					
		N monotherapy	0					

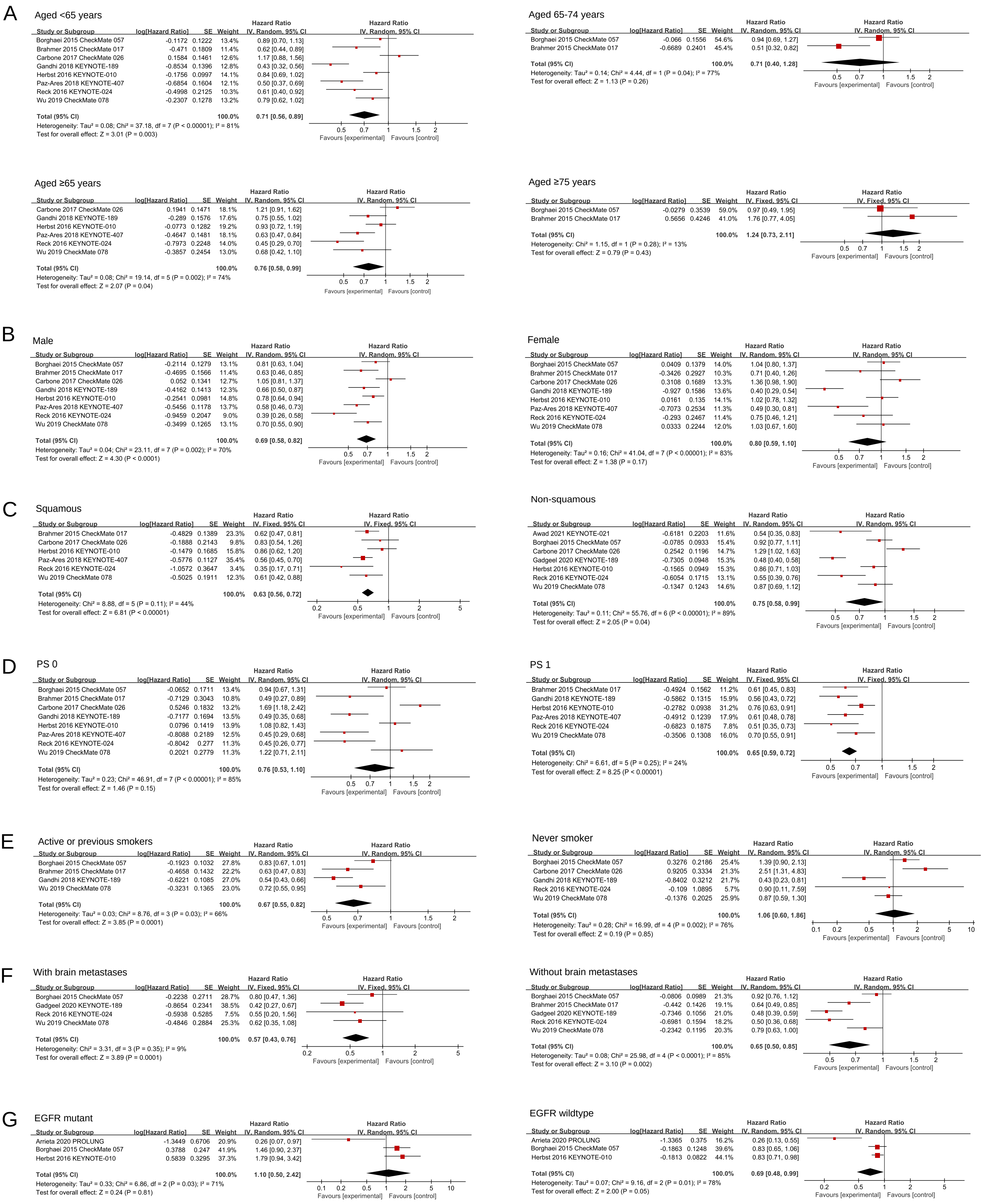
A
Aged <65 years
Study or Subgroup log[Hazard Ratio] SE Weight Hazard Ratio IV, Random, 95% CI
Borghaei 2015 CheckMate 057 -0.1172 0.1222 13.4% 0.89 [0.70, 1.13]
Brahmer 2015 CheckMate 017 -0.471 0.1809 11.4% 0.62 [0.44, 0.89]
Carbone 2017 CheckMate 026 0.1584 0.1461 12.6% 1.17 [0.88, 1.56]
Gandhi 2018 KEYNOTE-189 -0.8534 0.1396 12.8% 0.43 [0.32, 0.56]
Herbst 2016 KEYNOTE-010 -0.1756 0.0997 14.1% 0.84 [0.69, 1.02]
Paz-Ares 2018 KEYNOTE-407 -0.6854 0.1604 12.1% 0.50 [0.37, 0.69]
Reck 2016 KEYNOTE-024 -0.4998 0.2125 10.3% 0.61 [0.40, 0.92]
Wu 2019 CheckMate 078 -0.2307 0.1278 13.2% 0.79 [0.62, 1.02]
Total (95% CI) 100.0% 0.71 [0.56, 0.89]
Heterogeneity: Tau² = 0.08; Chi² = 37.18, df = 7 (P < 0.00001); I² = 81%
Test for overall effect: Z = 3.01 (P = 0.003)
0.5 0.7 1 1.5 2
Favours [experimental] Favours [control]
Aged 65-74 years
Study or Subgroup log[Hazard Ratio] SE Weight Hazard Ratio IV, Random, 95% CI
Borghaei 2015 CheckMate 057 -0.066 0.1556 54.6% 0.94 [0.69, 1.27]
Brahmer 2015 CheckMate 017 -0.6689 0.2401 45.4% 0.51 [0.32, 0.82]
Total (95% CI) 100.0% 0.71 [0.40, 1.28]
Heterogeneity: Tau² = 0.14; Chi² = 4.44, df = 1 (P = 0.04); I² = 77%
Test for overall effect: Z = 1.13 (P = 0.26)
0.5 0.7 1 1.5 2
Favours [experimental] Favours [control]
Aged ≥65 years
Study or Subgroup log[Hazard Ratio] SE Weight Hazard Ratio IV, Random, 95% CI
Carbone 2017 CheckMate 026 0.1941 0.1471 18.1% 1.21 [0.91, 1.62]
Gandhi 2018 KEYNOTE-189 -0.289 0.1576 17.6% 0.75 [0.55, 1.02]
Herbst 2016 KEYNOTE-010 -0.0773 0.1282 19.2% 0.93 [0.72, 1.19]
Paz-Ares 2018 KEYNOTE-407 -0.4647 0.1481 18.1% 0.63 [0.47, 0.84]
Reck 2016 KEYNOTE-024 -0.7973 0.2248 14.0% 0.45 [0.29, 0.70]
Wu 2019 CheckMate 078 -0.3857 0.2454 13.0% 0.68 [0.42, 1.10]
Total (95% CI) 100.0% 0.76 [0.58, 0.99]
Heterogeneity: Tau² = 0.08; Chi² = 19.14, df = 5 (P = 0.002); I² = 74%
Test for overall effect: Z = 2.07 (P = 0.04)
0.5 0.7 1 1.5 2
Favours [experimental] Favours [control]
Aged ≥75 years
Study or Subgroup log[Hazard Ratio] SE Weight Hazard Ratio IV, Fixed, 95% CI
Borghaei 2015 CheckMate 057 -0.0279 0.3539 59.0% 0.97 [0.49, 1.95]
Brahmer 2015 CheckMate 017 0.5656 0.4246 41.0% 1.76 [0.77, 4.05]
Total (95% CI) 100.0% 1.24 [0.73, 2.11]
Heterogeneity: Chi² = 1.15, df = 1 (P = 0.28); I² = 13%
Test for overall effect: Z = 0.79 (P = 0.43)
0.5 0.7 1 1.5 2
Favours [experimental] Favours [control]
B
Male
Study or Subgroup log[Hazard Ratio] SE Weight Hazard Ratio IV, Random, 95% CI
Borghaei 2015 CheckMate 057 -0.2114 0.1279 13.1% 0.81 [0.63, 1.04]
Brahmer 2015 CheckMate 017 -0.4695 0.1566 11.4% 0.63 [0.46, 0.85]
Carbone 2017 CheckMate 026 0.052 0.1341 12.7% 1.05 [0.81, 1.37]
Gandhi 2018 KEYNOTE-189 -0.4162 0.1413 12.3% 0.66 [0.50, 0.87]
Herbst 2016 KEYNOTE-010 -0.2541 0.0981 14.8% 0.78 [0.64, 0.94]
Paz-Ares 2018 KEYNOTE-407 -0.5456 0.1178 13.7% 0.58 [0.46, 0.73]
Reck 2016 KEYNOTE-024 -0.9459 0.2047 9.0% 0.39 [0.26, 0.58]
Wu 2019 CheckMate 078 -0.3499 0.1265 13.1% 0.70 [0.55, 0.90]
Total (95% CI) 100.0% 0.69 [0.58, 0.82]
Heterogeneity: Tau² = 0.04; Chi² = 23.11, df = 7 (P = 0.002); I² = 70%
Test for overall effect: Z = 4.30 (P < 0.0001)
0.5 0.7 1 1.5 2
Favours [experimental] Favours [control]
Female
Study or Subgroup log[Hazard Ratio] SE Weight Hazard Ratio IV, Random, 95% CI
Borghaei 2015 CheckMate 057 0.0409 0.1379 14.0% 1.04 [0.80, 1.37]
Brahmer 2015 CheckMate 017 -0.3426 0.2927 10.3% 0.71 [0.40, 1.26]
Carbone 2017 CheckMate 026 0.3108 0.1689 13.3% 1.36 [0.98, 1.90]
Gandhi 2018 KEYNOTE-189 -0.927 0.1586 13.6% 0.40 [0.29, 0.54]
Herbst 2016 KEYNOTE-010 0.0161 0.135 14.1% 1.02 [0.78, 1.32]
Paz-Ares 2018 KEYNOTE-407 -0.7073 0.2534 11.3% 0.49 [0.30, 0.81]
Reck 2016 KEYNOTE-024 -0.293 0.2467 11.4% 0.75 [0.46, 1.21]
Wu 2019 CheckMate 078 0.0333 0.2244 12.0% 1.03 [0.67, 1.60]
Total (95% CI) 100.0% 0.80 [0.59, 1.10]
Heterogeneity: Tau² = 0.16; Chi² = 41.04, df = 7 (P < 0.00001); I² = 83%
Test for overall effect: Z = 1.38 (P = 0.17)
0.5 0.7 1 1.5 2
Favours [experimental] Favours [control]
C
Squamous
Study or Subgroup log[Hazard Ratio] SE Weight Hazard Ratio IV, Fixed, 95% CI
Brahmer 2015 CheckMate 017 -0.4829 0.1389 23.3% 0.62 [0.47, 0.81]
Carbone 2017 CheckMate 026 -0.1888 0.2143 9.8% 0.83 [0.54, 1.26]
Herbst 2016 KEYNOTE-010 -0.1479 0.1685 15.8% 0.86 [0.62, 1.20]
Paz-Ares 2018 KEYNOTE-407 -0.5776 0.1127 35.4% 0.56 [0.45, 0.70]
Reck 2016 KEYNOTE-024 -1.0572 0.3647 3.4% 0.35 [0.17, 0.71]
Wu 2019 CheckMate 078 -0.5025 0.1911 12.3% 0.61 [0.42, 0.88]
Total (95% CI) 100.0% 0.63 [0.56, 0.72]
Heterogeneity: Chi² = 8.88, df = 5 (P = 0.11); I² = 44%
Test for overall effect: Z = 6.81 (P < 0.00001)
0.2 0.5 1 2 5
Favours [experimental] Favours [control]
Non-squamous
Study or Subgroup log[Hazard Ratio] SE Weight Hazard Ratio IV, Random, 95% CI
Awad 2021 KEYNOTE-021 -0.6181 0.2203 11.6% 0.54 [0.35, 0.83]
Borghaei 2015 CheckMate 057 -0.0785 0.0933 15.4% 0.92 [0.77, 1.11]
Carbone 2017 CheckMate 026 0.2542 0.1196 14.7% 1.29 [1.02, 1.63]
Gadgeel 2020 KEYNOTE-189 -0.7305 0.0948 15.3% 0.48 [0.40, 0.58]
Herbst 2016 KEYNOTE-010 -0.1565 0.0949 15.3% 0.86 [0.71, 1.03]
Reck 2016 KEYNOTE-024 -0.6054 0.1715 13.1% 0.55 [0.39, 0.76]
Wu 2019 CheckMate 078 -0.1347 0.1243 14.6% 0.87 [0.69, 1.12]
Total (95% CI) 100.0% 0.75 [0.58, 0.99]
Heterogeneity: Tau² = 0.11; Chi² = 55.76, df = 6 (P < 0.00001); I² = 89%
Test for overall effect: Z = 2.05 (P = 0.04)
0.5 0.7 1 1.5 2
Favours [experimental] Favours [control]
D
PS 0
Study or Subgroup log[Hazard Ratio] SE Weight Hazard Ratio IV, Random, 95% CI
Borghaei 2015 CheckMate 057 -0.0652 0.1711 13.4% 0.94 [0.67, 1.31]
Brahmer 2015 CheckMate 017 -0.7129 0.3043 10.8% 0.49 [0.27, 0.89]
Carbone 2017 CheckMate 026 0.5246 0.1832 13.2% 1.69 [1.18, 2.42]
Gandhi 2018 KEYNOTE-189 -0.7177 0.1694 13.5% 0.49 [0.35, 0.68]
Herbst 2016 KEYNOTE-010 0.0796 0.1419 13.9% 1.08 [0.82, 1.43]
Paz-Ares 2018 KEYNOTE-407 -0.8088 0.2189 12.5% 0.45 [0.29, 0.68]
Reck 2016 KEYNOTE-024 -0.8042 0.277 11.3% 0.45 [0.26, 0.77]
Wu 2019 CheckMate 078 0.2021 0.2779 11.3% 1.22 [0.71, 2.11]
Total (95% CI) 100.0% 0.76 [0.53, 1.10]
Heterogeneity: Tau² = 0.23; Chi² = 46.91, df = 7 (P < 0.00001); I² = 85%
Test for overall effect: Z = 1.46 (P = 0.15)
0.5 0.7 1 1.5 2
Favours [experimental] Favours [control]
PS 1
Study or Subgroup log[Hazard Ratio] SE Weight Hazard Ratio IV, Fixed, 95% CI
Brahmer 2015 CheckMate 017 -0.4924 0.1562 11.2% 0.61 [0.45, 0.83]
Gandhi 2018 KEYNOTE-189 -0.5862 0.1315 15.9% 0.56 [0.43, 0.72]
Herbst 2016 KEYNOTE-010 -0.2782 0.0938 31.2% 0.76 [0.63, 0.91]
Paz-Ares 2018 KEYNOTE-407 -0.4912 0.1239 17.9% 0.61 [0.48, 0.78]
Reck 2016 KEYNOTE-024 -0.6823 0.1875 7.8% 0.51 [0.35, 0.73]
Wu 2019 CheckMate 078 -0.3506 0.1308 16.0% 0.70 [0.55, 0.91]
Total (95% CI) 100.0% 0.65 [0.59, 0.72]
Heterogeneity: Chi² = 6.61, df = 5 (P = 0.25); I² = 24%
Test for overall effect: Z = 8.25 (P < 0.00001)
0.5 0.7 1 1.5 2
Favours [experimental] Favours [control]
E
Active or previous smokers
Study or Subgroup log[Hazard Ratio] SE Weight Hazard Ratio IV, Random, 95% CI
Borghaei 2015 CheckMate 057 -0.1923 0.1032 27.8% 0.83 [0.67, 1.01]
Brahmer 2015 CheckMate 017 -0.4658 0.1432 22.2% 0.63 [0.47, 0.83]
Gandhi 2018 KEYNOTE-189 -0.6221 0.1085 27.0% 0.54 [0.43, 0.66]
Wu 2019 CheckMate 078 -0.3231 0.1365 23.0% 0.72 [0.55, 0.95]
Total (95% CI) 100.0% 0.67 [0.55, 0.82]
Heterogeneity: Tau² = 0.03; Chi² = 8.76, df = 3 (P = 0.03); I² = 66%
Test for overall effect: Z = 3.85 (P = 0.0001)
0.5 0.7 1 1.5 2
Favours [experimental] Favours [control]
Never smoker
Study or Subgroup log[Hazard Ratio] SE Weight Hazard Ratio IV, Random, 95% CI
Borghaei 2015 CheckMate 057 0.3276 0.2186 25.4% 1.39 [0.90, 2.13]
Carbone 2017 CheckMate 026 0.9205 0.3334 21.3% 2.51 [1.31, 4.83]
Gandhi 2018 KEYNOTE-189 -0.8402 0.3212 21.7% 0.43 [0.23, 0.81]
Reck 2016 KEYNOTE-024 -0.109 1.0895 5.7% 0.90 [0.11, 7.59]
Wu 2019 CheckMate 078 -0.1376 0.2025 25.9% 0.87 [0.59, 1.30]
Total (95% CI) 100.0% 1.06 [0.60, 1.86]
Heterogeneity: Tau² = 0.28; Chi² = 16.99, df = 4 (P = 0.002); I² = 76%
Test for overall effect: Z = 0.19 (P = 0.85)
0.1 0.2 0.5 1 2 5 10
Favours [experimental] Favours [control]
F
With brain metastases
Study or Subgroup log[Hazard Ratio] SE Weight Hazard Ratio IV, Fixed, 95% CI
Borghaei 2015 CheckMate 057 -0.2238 0.2711 28.7% 0.80 [0.47, 1.36]
Gadgeel 2020 KEYNOTE-189 -0.8654 0.2341 38.5% 0.42 [0.27, 0.67]
Reck 2016 KEYNOTE-024 -0.5938 0.5285 7.5% 0.55 [0.20, 1.56]
Wu 2019 CheckMate 078 -0.4846 0.2884 25.3% 0.62 [0.35, 1.08]
Total (95% CI) 100.0% 0.57 [0.43, 0.76]
Heterogeneity: Chi² = 3.31, df = 3 (P = 0.35); I² = 9%
Test for overall effect: Z = 3.89 (P = 0.0001)
0.2 0.5 1 2 5
Favours [experimental] Favours [control]
Without brain metastases
Study or Subgroup log[Hazard Ratio] SE Weight Hazard Ratio IV, Random, 95% CI
Borghaei 2015 CheckMate 057 -0.0806 0.0989 21.3% 0.92 [0.76, 1.12]
Brahmer 2015 CheckMate 017 -0.442 0.1426 19.1% 0.64 [0.49, 0.85]
Gadgeel 2020 KEYNOTE-189 -0.7346 0.1056 21.0% 0.48 [0.39, 0.59]
Reck 2016 KEYNOTE-024 -0.6981 0.1594 18.2% 0.50 [0.36, 0.68]
Wu 2019 CheckMate 078 -0.2342 0.1195 20.3% 0.79 [0.63, 1.00]
Total (95% CI) 100.0% 0.65 [0.50, 0.85]
Heterogeneity: Tau² = 0.08; Chi² = 25.98, df = 4 (P < 0.0001); I² = 85%
Test for overall effect: Z = 3.10 (P = 0.002)
0.5 0.7 1 1.5 2
Favours [experimental] Favours [control]
G
EGFR mutant
Study or Subgroup log[Hazard Ratio] SE Weight Hazard Ratio IV, Random, 95% CI
Arrieta 2020 PROLUNG -1.3449 0.6706 20.9% 0.26 [0.07, 0.97]
Borghaei 2015 CheckMate 057 0.3788 0.247 41.9% 1.46 [0.90, 2.37]
Herbst 2016 KEYNOTE-010 0.5839 0.3295 37.3% 1.79 [0.94, 3.42]
Total (95% CI) 100.0% 1.10 [0.50, 2.42]
Heterogeneity: Tau² = 0.33; Chi² = 6.86, df = 2 (P = 0.03); I² = 71%
Test for overall effect: Z = 0.24 (P = 0.81)
0.1 0.2 0.5 1 2 5 10
Favours [experimental] Favours [control]
EGFR wildtype
Study or Subgroup log[Hazard Ratio] SE Weight Hazard Ratio IV, Random, 95% CI
Arrieta 2020 PROLUNG -1.3365 0.375 16.2% 0.26 [0.13, 0.55]
Borghaei 2015 CheckMate 057 -0.1863 0.1248 39.6% 0.83 [0.65, 1.06]
Herbst 2016 KEYNOTE-010 -0.1813 0.0822 44.1% 0.83 [0.71, 0.98]
Total (95% CI) 100.0% 0.69 [0.48, 0.99]
Heterogeneity: Tau² = 0.07; Chi² = 9.16, df = 2 (P = 0.01); I² = 78%
Test for overall effect: Z = 2.00 (P = 0.05)
0.2 0.5 1 2 5
Favours [experimental] Favours [control]

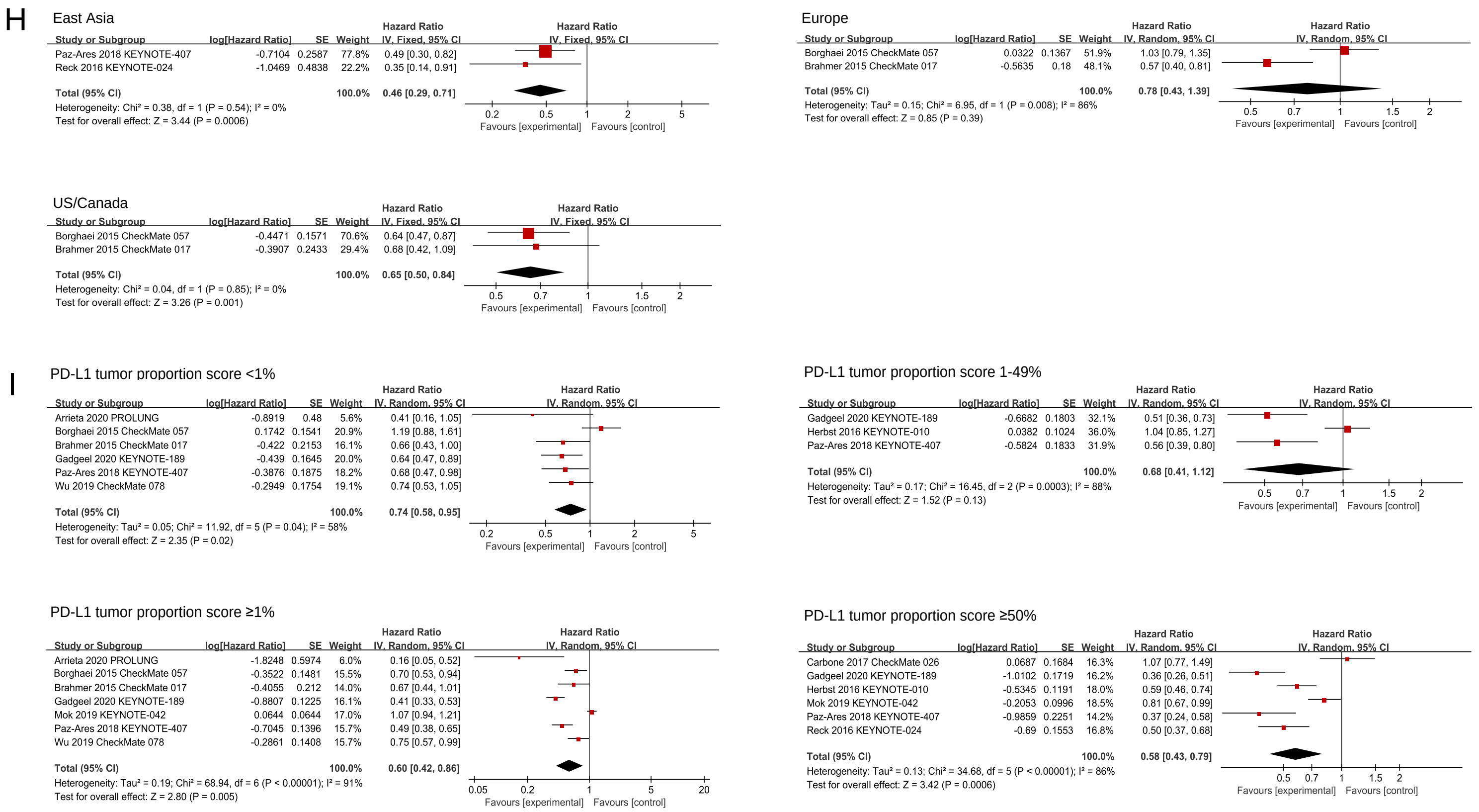

Figure S1: Forest plots of HRs comparing PFS between PD-1 inhibitors based therapy and non PD-1 inhibitor based therapy with respect to (A) age group, (B) gender, (C) histomorphological subtypes, (D) PS score, (E) smoking status, (F) metastases status, (G) EGFR mutation status, (H) region and (I) PD-L1 tumor proportion score.

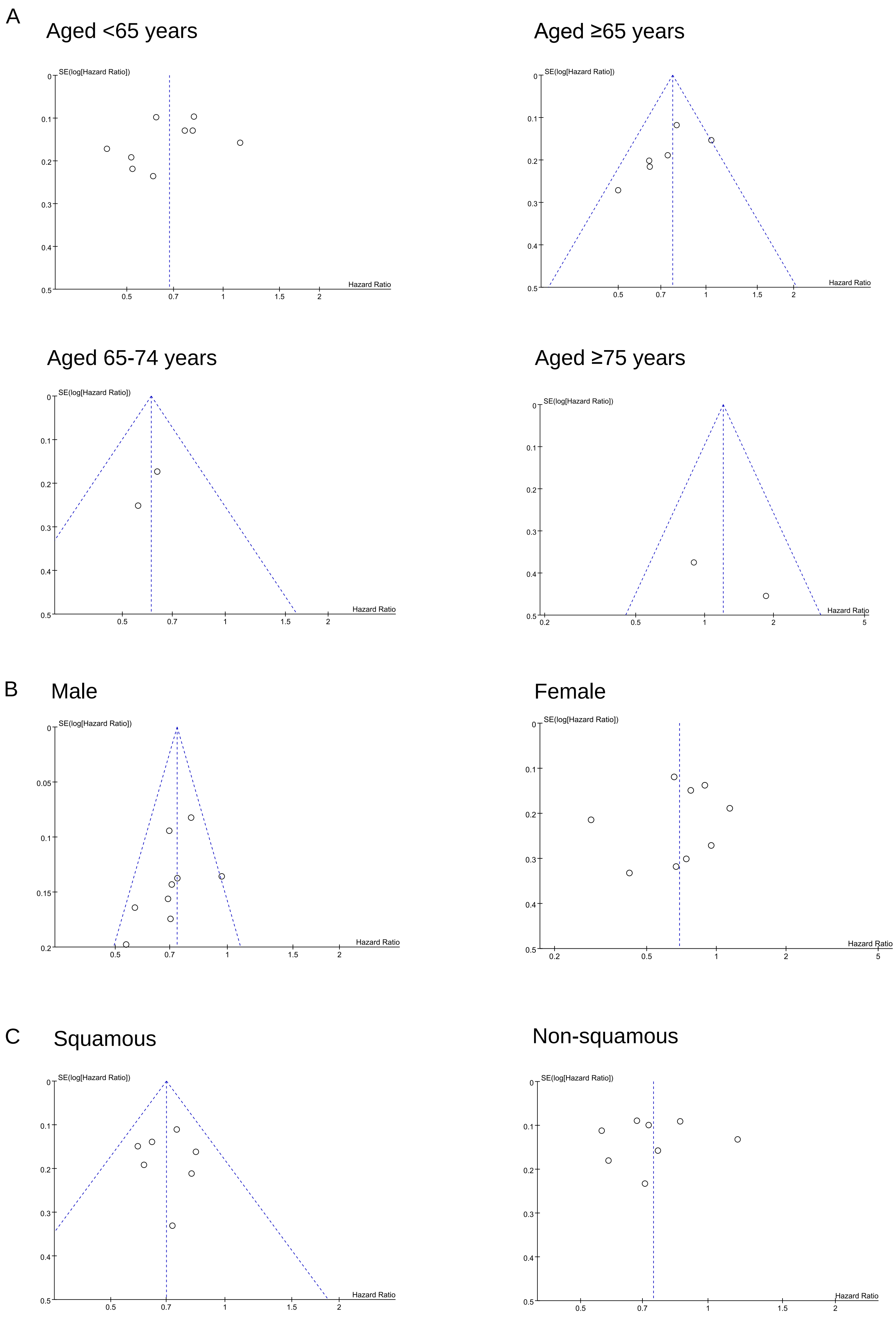
A
Aged <65 years
SE(log[Hazard Ratio])
Hazard Ratio
Aged ≥65 years
SE(log[Hazard Ratio])
Hazard Ratio
Aged 65-74 years
SE(log[Hazard Ratio])
Hazard Ratio
Aged ≥75 years
SE(log[Hazard Ratio])
Hazard Ratio
B
Male
SE(log[Hazard Ratio])
Hazard Ratio
Female
SE(log[Hazard Ratio])
Hazard Ratio
C
Squamous
SE(log[Hazard Ratio])
Hazard Ratio
Non-squamous
SE(log[Hazard Ratio])
Hazard Ratio

D

PS 0

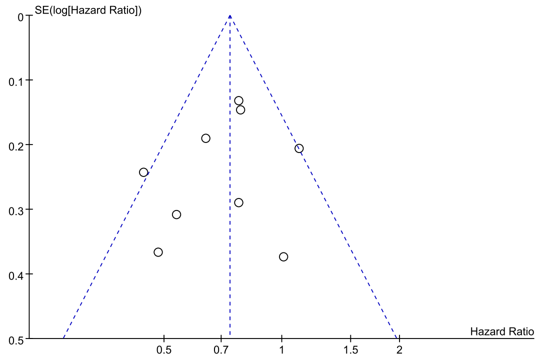

PS 1

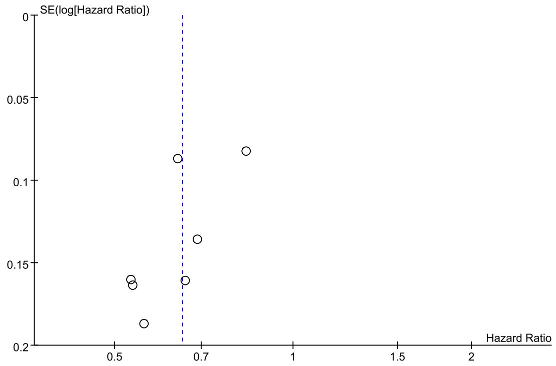

E

Active or previous smokers

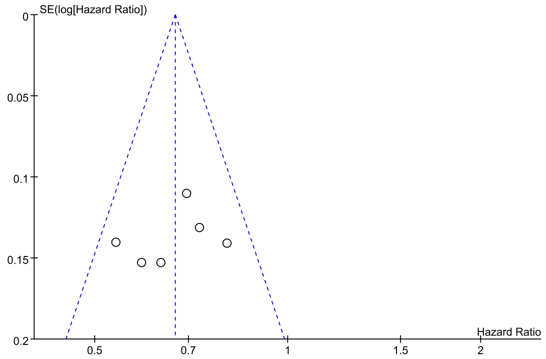

Never smoker

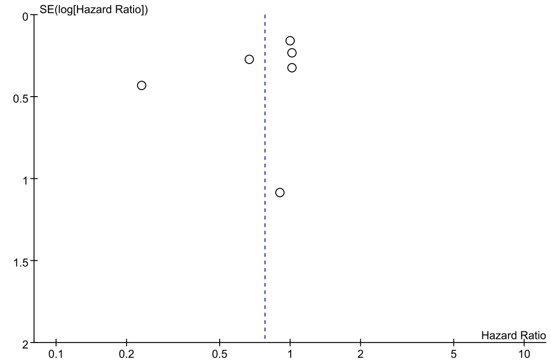

F

With brain metastases

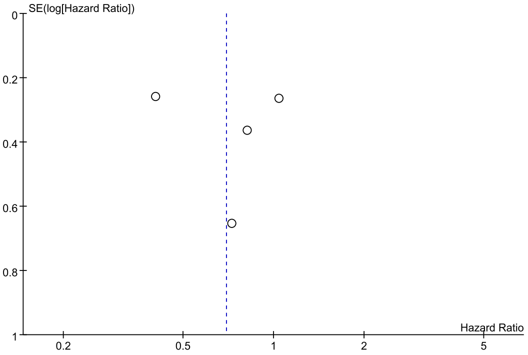

Without brain metastases

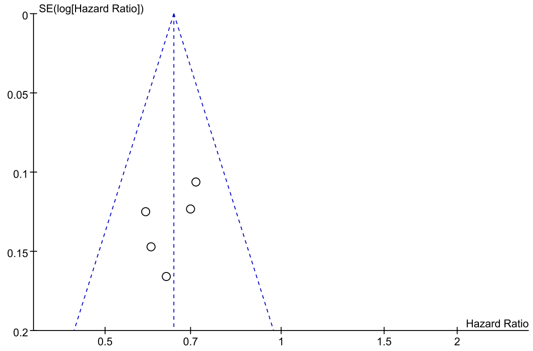

Liver metastases

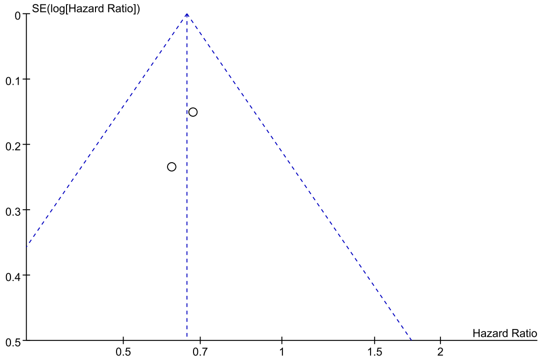

G

EGFR mutant

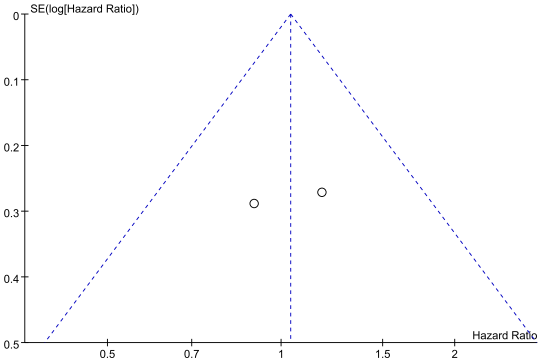

EGFR wildtype

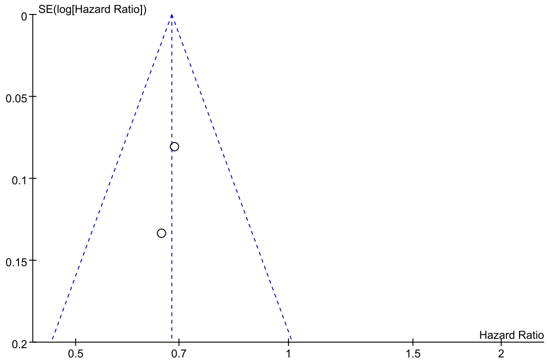

H

East Asia

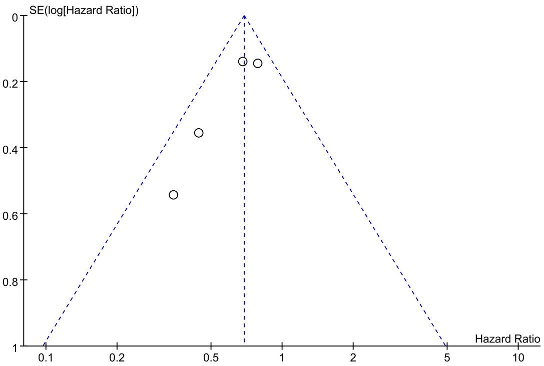

Europe

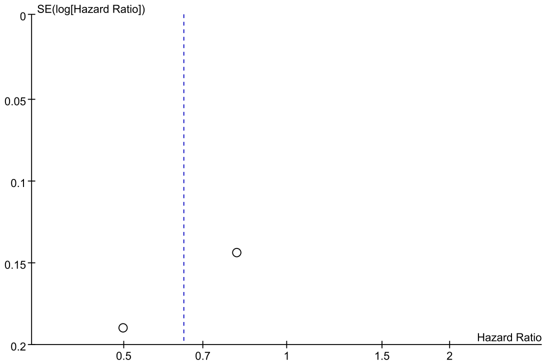

US/Canada

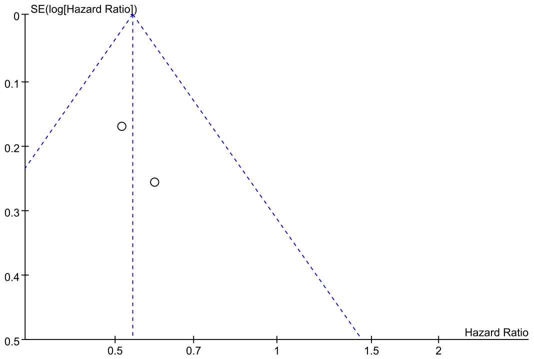

I

PD-L1 tumor proportion score <1%

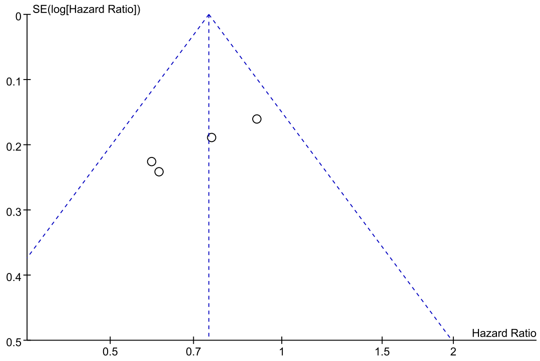

PD-L1 tumor proportion score ≥1%

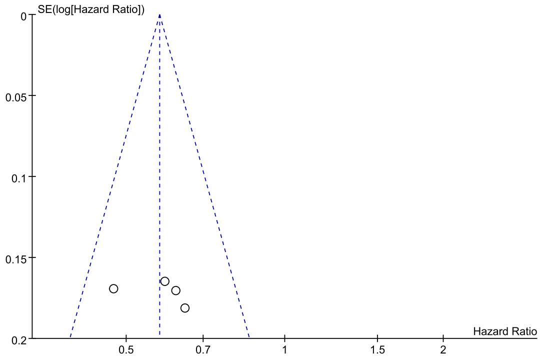

PD-L1 tumor proportion score 1-49%

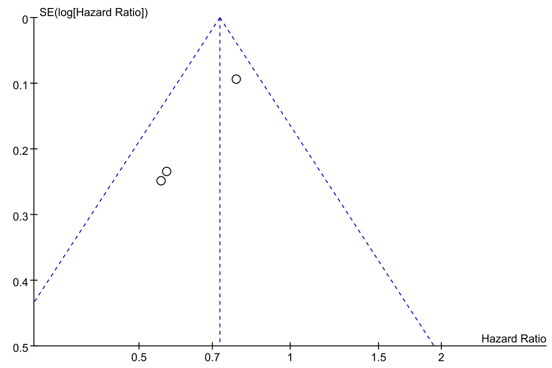

PD-L1 tumor proportion score ≥50%

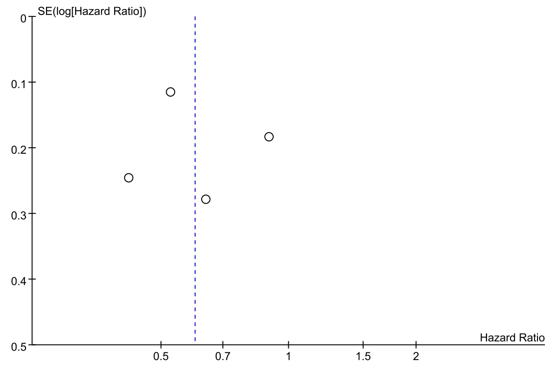

Figure S2: Funnel plots for OS in the subgroup with respect to (A) age group, (B) gender, (C) histomorphological subtypes, (D) PS score, (E) smoking status, (F) metastases status/site, (G) EGFRmutation status, (H) region and (I) PD-L1tumor proportion score.

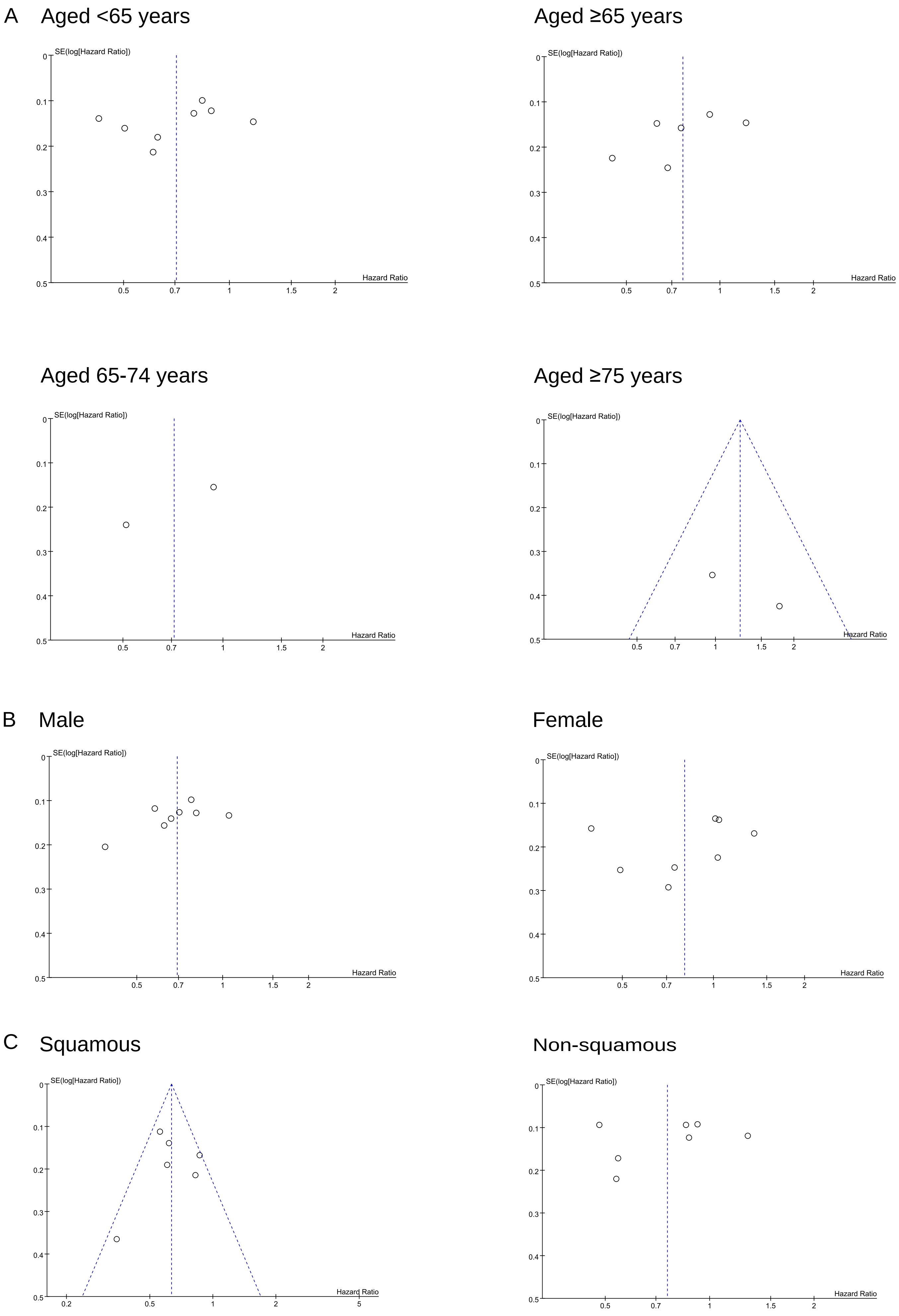
A
Aged <65 years
SE(log[Hazard Ratio])
Hazard Ratio
Aged ≥65 years
SE(log[Hazard Ratio])
Hazard Ratio
Aged 65-74 years
SE(log[Hazard Ratio])
Hazard Ratio
Aged ≥75 years
SE(log[Hazard Ratio])
Hazard Ratio
B
Male
SE(log[Hazard Ratio])
Hazard Ratio
Female
SE(log[Hazard Ratio])
Hazard Ratio
C
Squamous
SE(log[Hazard Ratio])
Hazard Ratio
Non-squamous
SE(log[Hazard Ratio])
Hazard Ratio

D PS 0

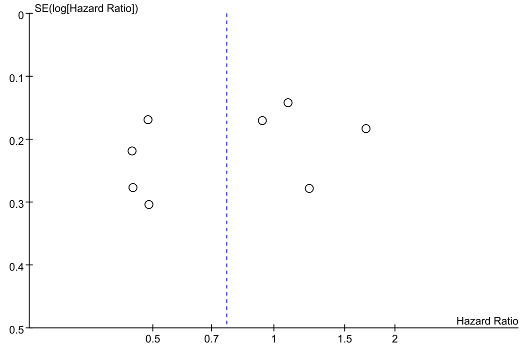

PS 1

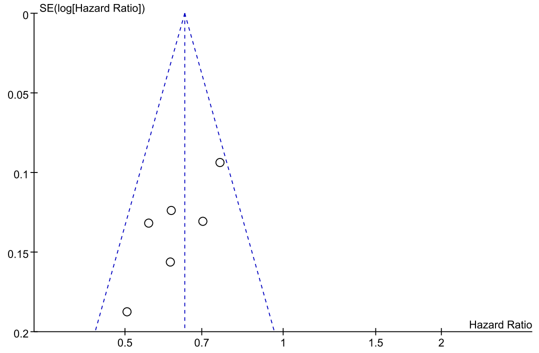

E Active or previous smokers

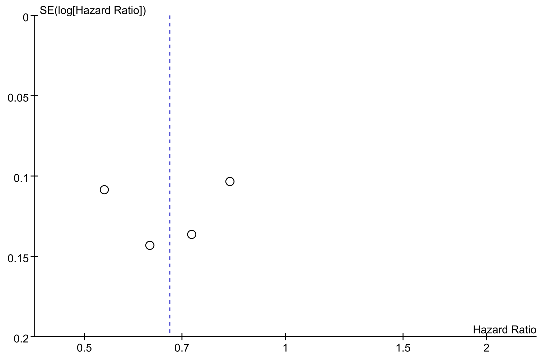

Never smoker

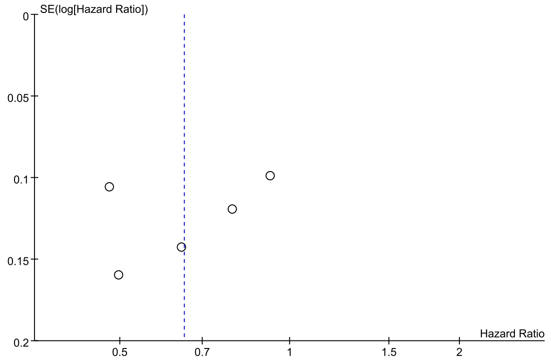

F With brain metastases

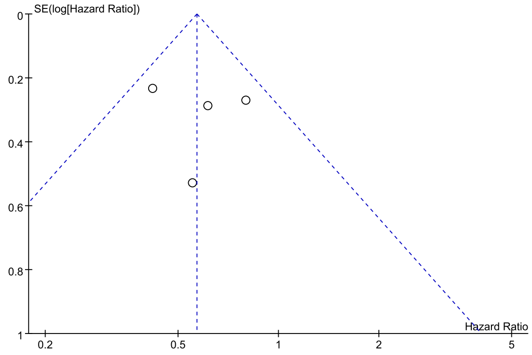

Without brain metastases

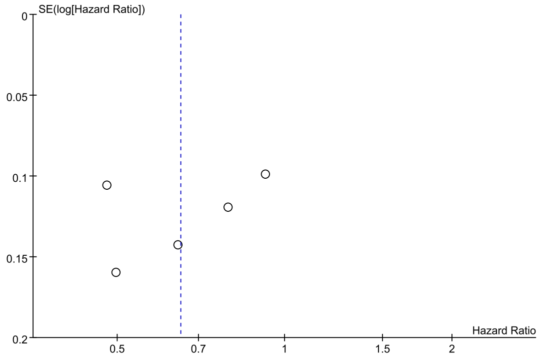

G EGFR mutant

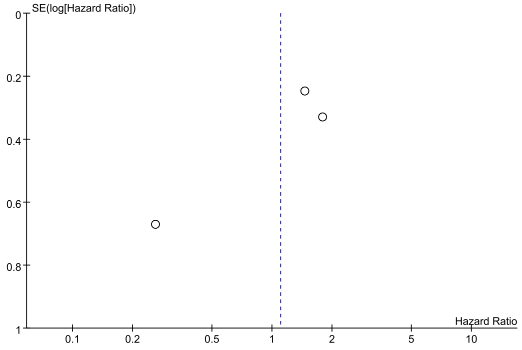

EGFR wildtype

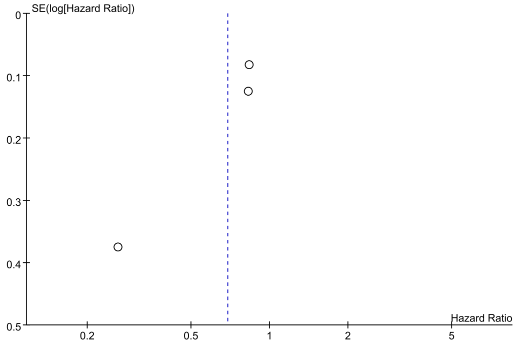

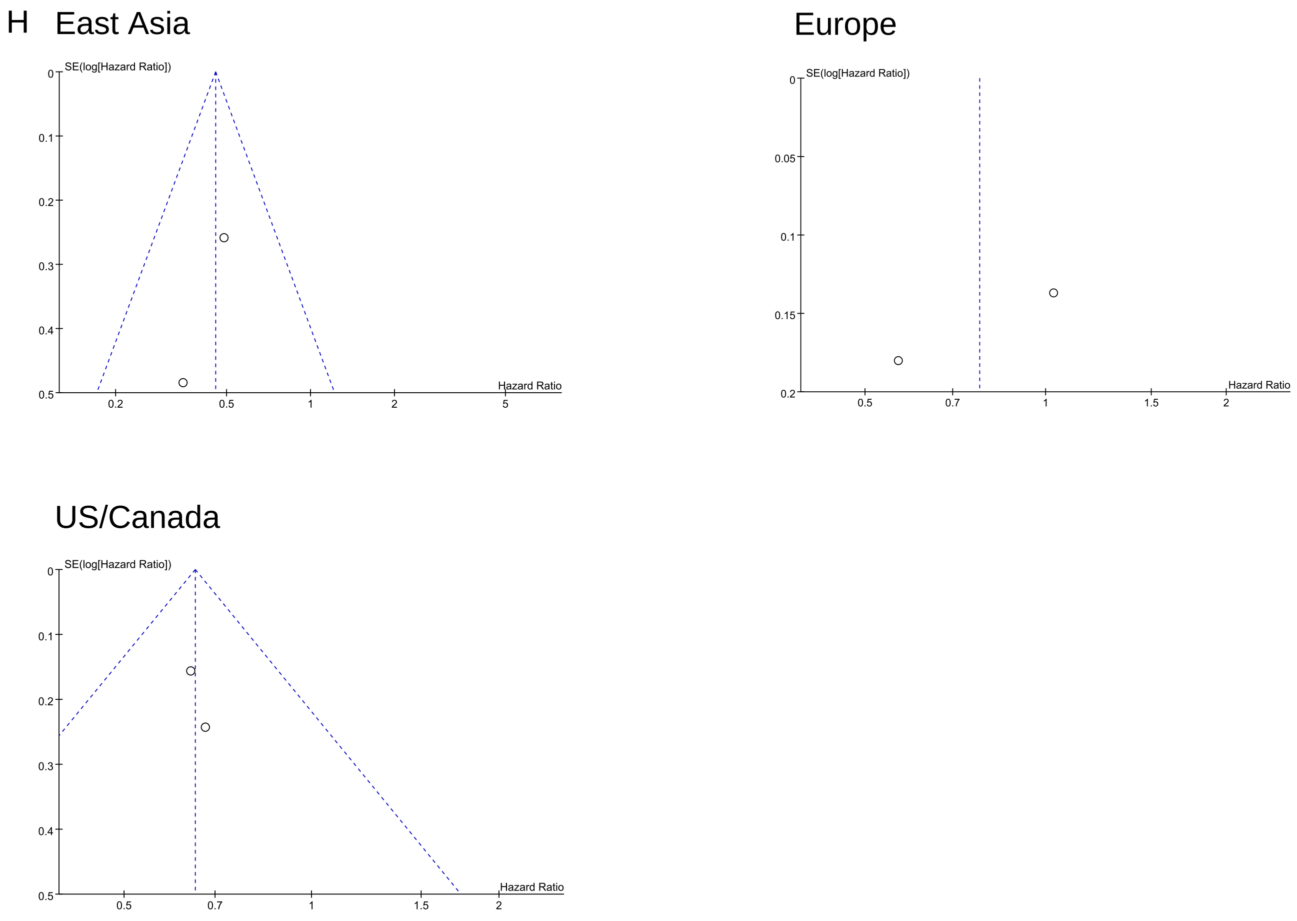

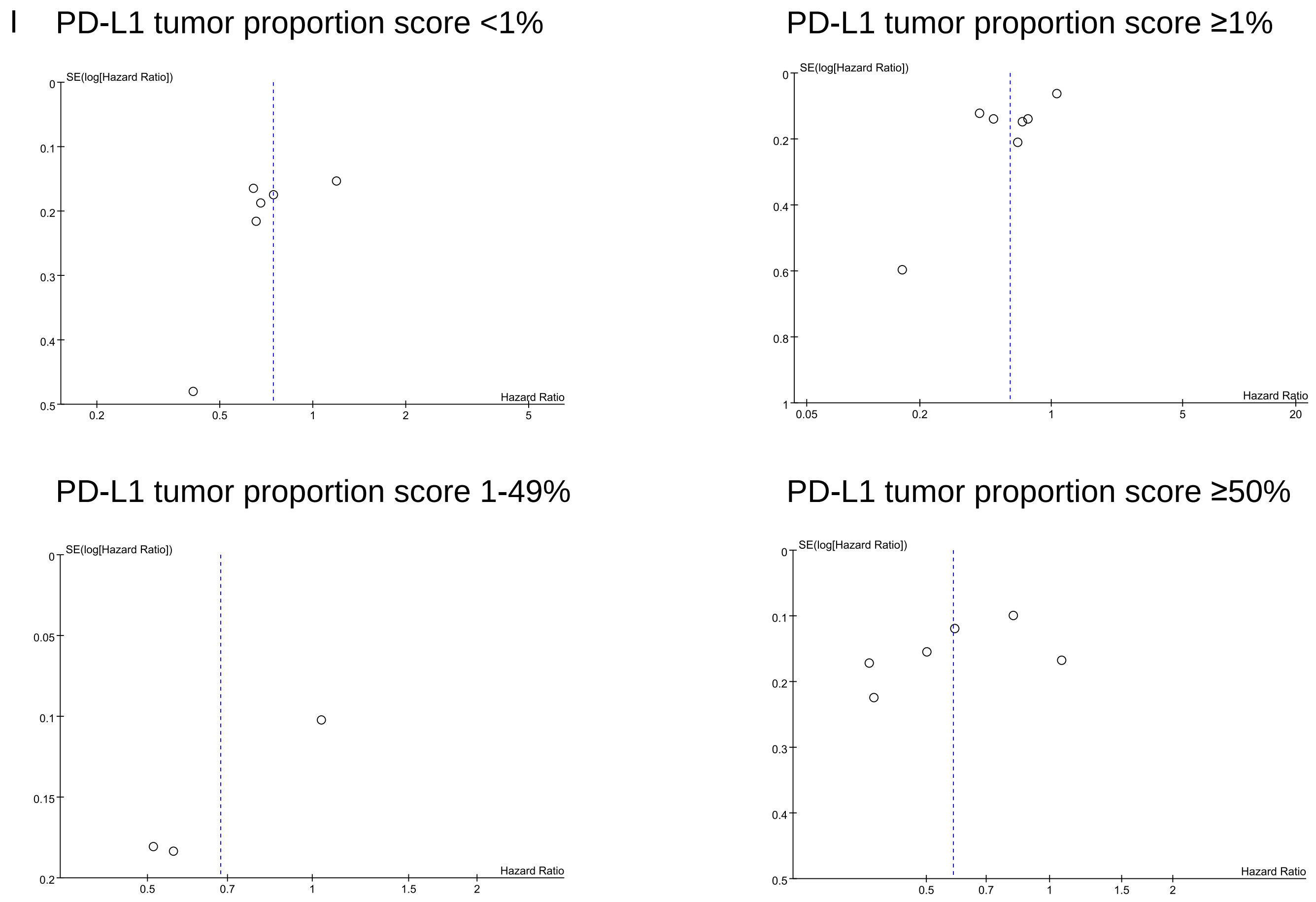

Figure S3: Funnel plots for PFS in the subgroup with respect to (A) age group, (B) gender, (C) histomorphological subtypes, (D) PS score, (E) smoking status, (F) metastases status, (G) EGFRmutation status, (H) region and (I) PD-L1tumor proportion score.
